# Liver kinase B1 maintains natural killer cell survival by regulating redox homeostasis

**DOI:** 10.1038/s41419-026-08629-w

**Published:** 2026-03-27

**Authors:** Wanqing Meng, Liang Luo, Zhiqiang Xiao, Jiayuan Huang, Yong Huang, Mingyue Zhao, Wenkai Lv, Bing Xin, Peiran Feng, Jun He, Hanlin Shuai, Zhongjun Dong, Meixiang Yang

**Affiliations:** 1 Guangdong Provincial Key Laboratory of Spine and Spinal Cord Reconstruction, The Fifth Affiliated Hospital of Jinan University (Heyuan Shenhe People’s Hospital), Jinan University, Heyuan, 517000 China; 2 The Affiliated Guangdong Second Provincial General Hospital of Jinan University, Jinan University, Guangzhou, 510317 China; 3 State Key Laboratory of Bioactive Molecules and Druggability Assessment, The Biomedical Translational Research Institute, Health Science Center (School of Medicine), Jinan University, Guangzhou, 510632 China; 4 Peking University Shenzhen Hospital, Department of Pathology, Shenzhen Peking University-The Hong Kong University of Science and Technology Medical Center, Shenzhen, 518036 China; 5 Key Laboratory of Ministry of Education for Viral Pathogenesis & Infection Prevention and Control (Jinan University), Guangzhou Key Laboratory for Germ-free Animals and Microbiota Application, Institute of Laboratory Animal Science, School of Medicine, Jinan University, Guangzhou, 510632 China; 6 The First Affiliated Hospital of Anhui Medical University and Institute for Clinical Immunology, Anhui Medical University, Hefei, 230032 China; 7 School of Medicine and Institute for Immunology, Beijing Key Lab for Immunological Research on Chronic Diseases, Tsinghua University, Beijing, 100084 China

**Keywords:** Immune cell death, Apoptosis

## Abstract

Natural killer (NK) cells are the primary innate lymphoid cells responsible for antiviral defense and tumor immunosurveillance. However, further clarification is needed on how to prevent their over-activation and maintain their quiescence during development. In this study, we present evidence that liver kinase B1 (Lkb1) functions as a critical metabolic checkpoint, regulating NK cell survival and preserving their effective tumor immunosurveillance capabilities. Genetic ablation of Lkb1 led to mitochondrial dysfunction and impaired autophagy, resulting in reactive oxygen species (ROS)-dependent cell death. Additionally, Lkb1 deficiency disrupted iron homeostasis, causing iron overload and the subsequent accumulation of cytotoxic lipid ROS. Targeted interventions aimed at inhibiting ROS accumulation or iron overload significantly rescued the survival defect in Lkb1-deficient NK cells. Notably, these regulatory functions could not be rescued by pharmacologic AMPK activation or mTORC1 inhibition. Furthermore, the deletion of Lkb1 increased the expression of inhibitory receptors PD-1 and TIGIT, further impairing NK cell-mediated tumor surveillance. Our investigation collectively highlights the critical role of Lkb1 in maintaining NK cell quiescence through the coordinated regulation of metabolic fitness and redox balance, offering new insights into the metabolic programming of NK cell development and function.

## Introduction

Natural killer (NK) cells serve as critical components of innate immunity, playing essential roles in antiviral defense and tumor surveillance, as well as mediating crosstalk between innate and adaptive immune responses through cytokine production and cellular interactions [1–3]. Originating from common lymphoid progenitors, NK cells undergo a precisely regulated developmental process spanning both bone marrow and peripheral tissues [4, 5]. The maintenance of homeostasis and functionality in NK cells relies on the precise regulation of various intracellular and extracellular biochemical signals. Among these, IL-15 is a master regulator of NK cell biology, governing lineage commitment and terminal maturation through activation of JAK-STAT and PI3K-mTOR signaling pathways [6, 7]. Disruptions in these cascades have been shown to impair NK cell homeostasis and effector functions [8–14], underscoring the importance of metabolic regulation in NK cell biology. The quiescent homeostasis of immune cells is of great biological significance as it prevents cells from prematurely activating or being exhausted, thus preserving their capacity to respond effectively to pathogens and tumors [15–17]. Although recent breakthroughs have deepened our comprehension of immunometabolism, the inhibitory pathways that restrict NK cell metabolism and sustain the quiescent state are not well understood.

Liver kinase B1 (Lkb1), encoded by the *Stk11* gene, is a conserved serine/threonine kinase that serves as a central regulator of cellular metabolism and survival. By activating downstream AMPK-family kinases, Lkb1 orchestrates energy homeostasis to coordinate cell growth, survival, and metabolic reprogramming [18, 19]. Emerging evidence highlights its pleiotropic roles in immune regulation, modulating functional states and fate decisions across diverse immune cell types [20–28]. Interestingly, studies have revealed that Lkb1 primarily regulates immune cell development and function through pathways independent of AMPK-mTOR signaling [20, 21, 28]. Our recent study demonstrated that Lkb1 is essential for maintaining γδ T cell function and preventing IL-17^+^ γδ T cell-mediated autoimmune hepatitis [29]. However, the role of Lkb1 in NK cell developmental progression, metabolic adaptation, and effector function remains unexplored. Understanding these aspects may reveal new strategies to enhance NK cell-based immunotherapies and improve outcomes in infectious diseases and cancer.

To investigate the physiological function of Lkb1 in NK cells, we established three complementary genetic mouse models: early/late stage-specific Lkb1 knockout and an inducible Lkb1 deletion system. Our results demonstrated that Lkb1 is vital for NK cell homeostasis, suppressing excessive apoptosis and metabolic hyperactivity. Genetic ablation of Lkb1 led to significant NK cell lymphopenia and functional impairment in vivo. Lkb1 deficiency triggered a cascade of metabolic disturbances, including hyperactive mitochondrial respiration, impaired autophagy, and excessive ROS accumulation, collectively disrupting redox balance and promoting cell death. Notably, Lkb1 maintains iron homeostasis in NK cells, and its loss resulted in iron dysregulation and lipid peroxidation. The combined treatment with the iron chelator deferoxamine (DFO) and the pan-caspase inhibitor Z-VAD-FMK synergistically reduced cell death. These findings highlight Lkb1 as a key regulator of NK cell survival and function, providing new insights into innate immunity and immune surveillance.

## Results

### Deletion of Lkb1 results in severe NK cell lymphopenia and developmental arrest

**Fig. 1:**
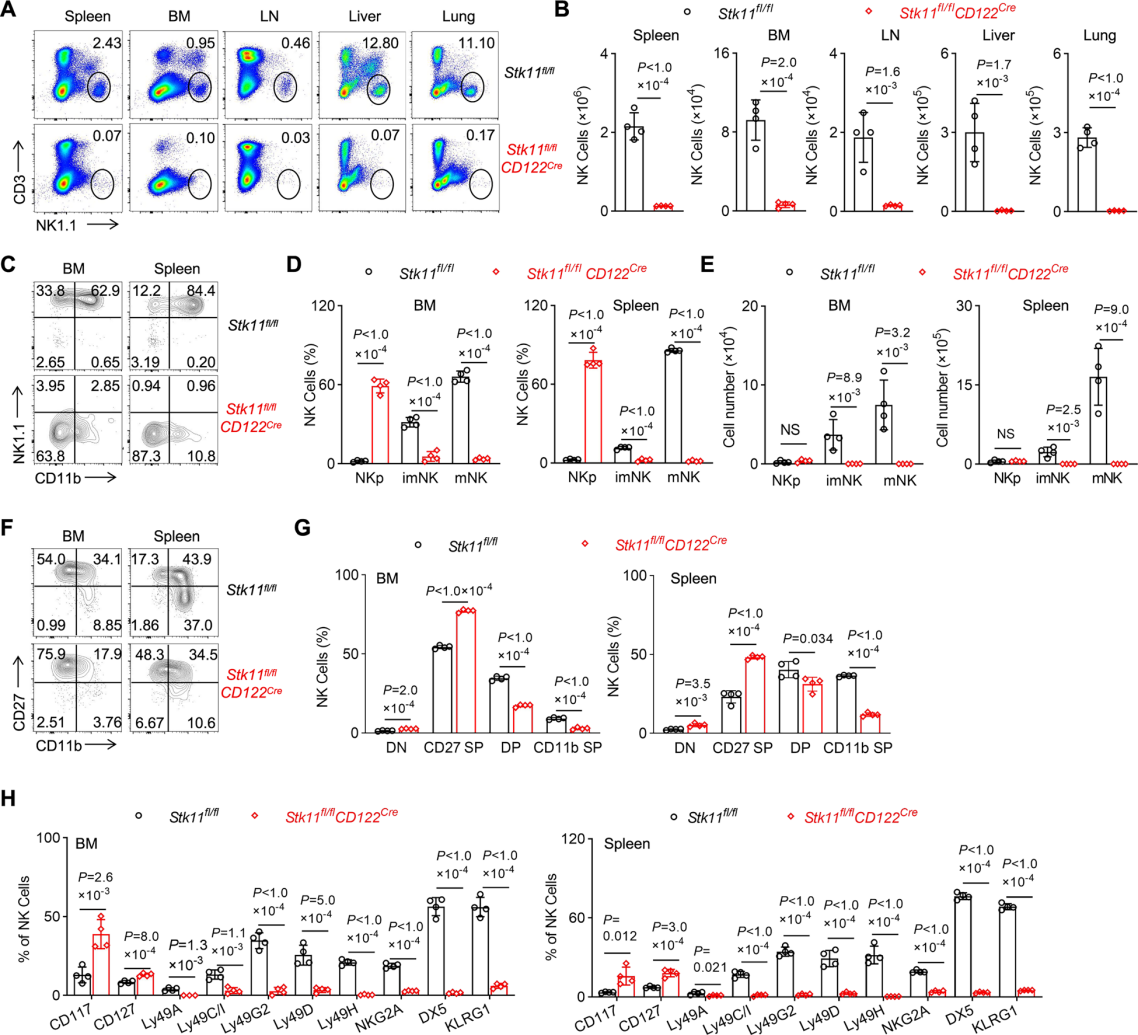
Genetic deletion of Lkb1 at the NKp stage results in severe NK cell lymphopenia and developmental arrest. **A**, **B** Representative flow cytometry plots (**A**) and absolute number quantification (**B**) of NK cells (CD3^−^NK1.1^+^) in the spleen, bone marrow (BM), lymph nodes (LN), liver, and lung from *Stk11*^*fl/fl*^ and *Stk11*^*fl/fl*^*CD122*^*Cre*^ mice (*n* = 4). **C**–**E** Representative flow cytometry plots (**C**), percentages (**D**), and absolute number quantification (**E**) of NKp, imNK, and mNK cells in the BM and spleen from *Stk11*^*fl/fl*^ and *Stk11*^*fl/fl*^*CD122*^*Cre*^ mice (*n* = 4). **F**, **G** Representative flow cytometry plots (**F**) and percentages (**G**) of DN, CD27 SP, DP, and CD11b SP cells within gated CD3^−^NKp46^+^ cells in the BM (left) and spleen (right) from *Stk11*^*fl/fl*^ and *Stk11*^*fl/fl*^*CD122*^*Cre*^ mice (*n* = 4). **H** Percentages of developmental-related markers expressed on CD3^−^NK1.1^+^ NK cells isolated from the BM (left) and spleen (right) of *Stk11*^*fl/fl*^ and *Stk11*^*fl/fl*^*CD122*^*Cre*^ mice (*n* = 4). Data are presented as mean ± standard deviation (SD). Two-tailed Student’s *t* test (**B**, **D**, **E**, **G**, **H**); NS, not significant. Data are representative of three (**A**–**G**) or two (**H**) independent experiments.

To investigate the essential role of Lkb1 in the development and homeostasis of NK cells, we generated *Stk11*^*fl/fl*^*CD122*^*Cre*^ mice to achieve stage-specific *Lkb1* ablation at the NK progenitor (NKp) stage (Fig. S1A). Quantitative PCR analysis confirmed efficient *Lkb1* deletion, showing markedly reduced *Stk11* transcript levels across all NK cell subsets compared to wild-type (WT) controls (Fig. S1B). Given that Lkb1 is crucial for cellular metabolism and growth, we first analyzed the quantity and development process of NK cells using flow cytometry (Fig. S2A). Systematic immunophenotyping revealed that Lkb1 deficiency induced severe systemic NK cell lymphopenia, manifesting as significantly diminished frequencies and absolute counts in multiple organs, including the spleen, bone marrow (BM), lymph nodes (LN), liver, and lung (Fig. 1A, B). Developmental profiling uncovered a striking accumulation of NKp cells, accompanied by reduced proportions and absolute numbers of both immature (iNK) and mature (mNK) NK cell subsets (Fig. 1C–E). The developmental blockade was further corroborated by CD27/CD11b-based subset analysis, which showed an enrichment of CD27^−^CD11b^−^ double-negative (DN) cells and CD27^+^CD11b^−^ iNK populations, alongside a dramatic contraction of CD27^+^CD11b^+^ and CD27^−^CD11b^+^ mature subsets in Lkb1-deficient mice (Fig. 1F, G). Surface marker characterization revealed that Lkb1-null NK cells maintained elevated expression of progenitor markers (CD117 and CD127) while exhibiting significantly reduced levels of maturation markers, including Ly49 receptors and KLRG1, in both BM and spleen (Fig. 1H). Together, these data demonstrate that Lkb1 ablation at the NKp stage creates a developmental obstacle, severely impairing the NKp-to-iNK transition and subsequent terminal maturation. These findings further imply that Lkb1 serves as an indispensable metabolic regulator governing critical checkpoints in NK cell development and population maintenance.

### Lkb1 governs NK cell homeostasis in a cell-intrinsic manner

**Fig. 2:**
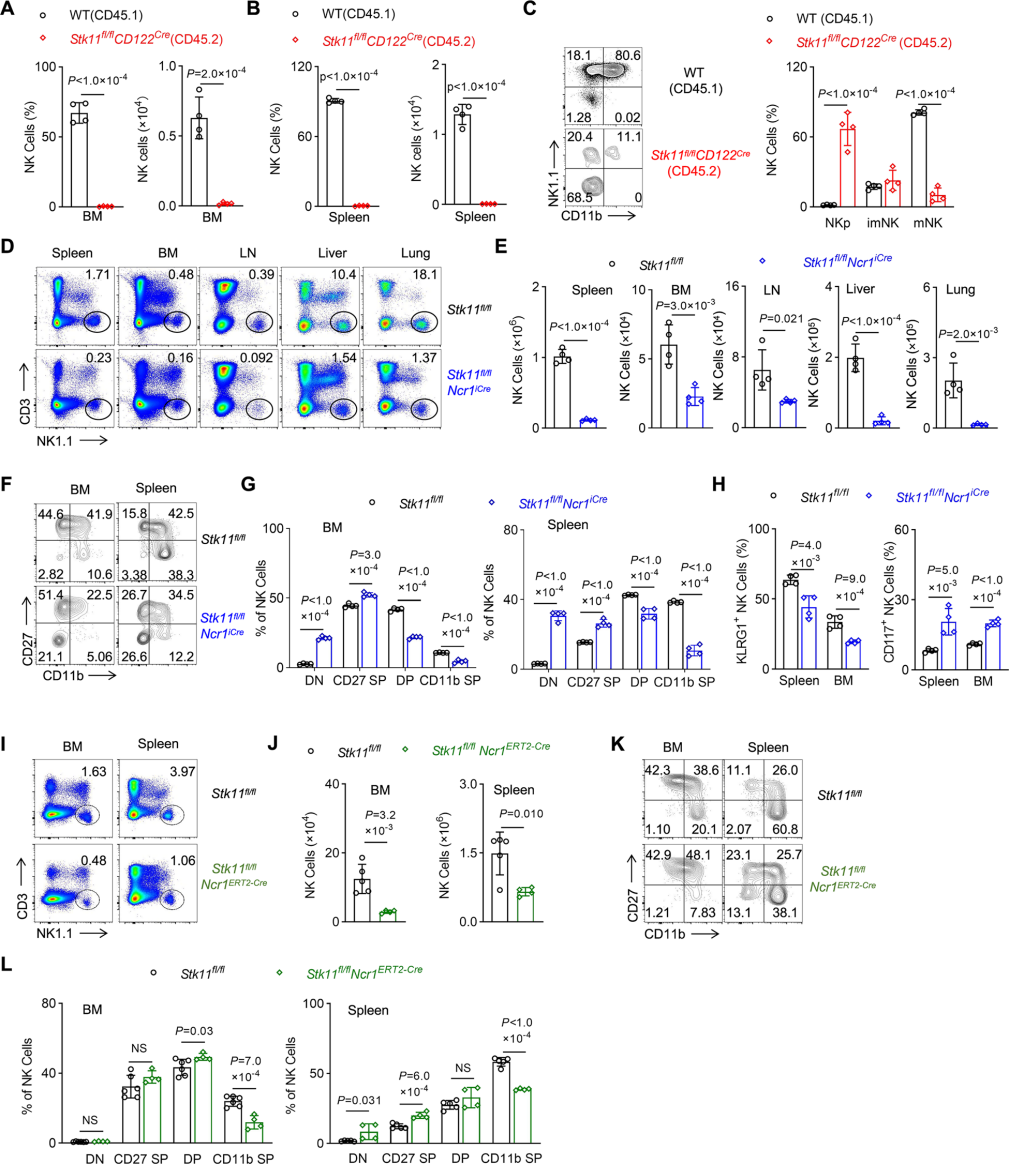
Lkb1 regulates NK cell homeostasis in a cell-intrinsic manner. **A**, **B** The proportion (left) and absolute number (right) of NK cells in the bone marrow (**A**) and spleen (**B**) from recipient mice after 8 weeks of reconstruction (*n* = 4). **C** Representative flow cytometry plots (left) and the percentage (right) of NKp, imNK, and mNK cells on gated CD3^−^CD122^+^ cells in CD45.1^+^ or CD45.2^+^ splenocytes from recipient mice after 8 weeks of reconstruction (*n* = 4). **D**, **E** Representative flow cytometry plots (**D**) and quantification of the absolute number (**E**) of NK cells (CD3^−^NK1.1^+^) in the spleen, bone marrow, lymph nodes, liver, and lung from *Stk11*^*fl/fl*^ and *Stk11*^*fl/fl*^*Ncr1*^*iCre*^ mice (*n* = 4). **F**, **G** Representative flow cytometry plots (**F**) and the percentages (**G**) of DN, CD27 SP, DP, and CD11b SP cells on gated CD3^−^NKp46^+^ cells in the bone marrow (**G**, left) and spleen (**G**, right) from *Stk11*^*fl/fl*^ and *Stk11*^*fl/fl*^*Ncr1*^*iCre*^ mice (*n* = 4). **H** The percentages of KLRG1^+^ (left) and CD117^+^ (right) cells on gated CD3^−^NK1.1^+^ cells in the spleen and BM from *Stk11*^*fl/fl*^ and *Stk11*^*fl/fl*^*Ncr1*^*iCre*^ mice (*n* = 4). **I**, **J** Representative flow cytometry plots (**I**) and quantification of the absolute number (**J**) of NK cells (CD3^−^NK1.1^+^) in the BM and spleen from tamoxifen-induced *Stk11*^*fl/fl*^ and *Stk11*^*fl/fl*^*Ncr1*^*ERT2-Cre*^ mice (*n* = 4). **K**, **L** Representative flow cytometry plots (**K**) and the percentages (**L**) of DN, CD27 SP, DP, and CD11b SP cells on gated CD3^−^NKp46^+^ cells in the BM and spleen of tamoxifen-induced *Stk11*^*fl/fl*^ and *Stk11*^*fl/fl*^*Ncr1*^*ERT2-Cre*^ mice (*n* = 4). Data are shown as mean ± SD. Two-tailed Student’s *t* test (**A**–**C**, **E**, **G**, **H**, **J**, **L**); NS, not significant. Data are representative of two (**A**, **B**) or three (**C**–**L**) independent experiments.

To investigate the cell-intrinsic role of Lkb1 in NK cell development, we utilized three distinct mouse models. Initially, competitive bone marrow chimera assays were conducted by co-transferring bone marrow cells from *Stk11*^*fl/fl*^*CD122*^*Cre*^ mice (CD45.2) and WT mice (CD45.1) at a 1:1 ratio into irradiated CD45.1^+^CD45.2^+^ recipients (Fig. S3A). Quantitative analysis revealed an impaired reconstitution capacity of Lkb1-deficient hematopoietic progenitors, with significantly reduced contributions to the BM and spleen NK cell compartments compared to WT counterparts (Figs. 2A, B, S3B). Developmental profiling indicated that NK cells derived from *Stk11*^*fl/fl*^*CD122*^*Cre*^ bone marrow were predominantly arrested in the NKp stage, accompanied by a marked decrease in the proportion of mature NK (mNK) cells (Fig. 2C). To confirm NK cell-specific effects, we generated *Stk11*^*fl/fl*^*Ncr1*^*iCre*^ mice with selective *Lkb1* deletion in NK cells (Fig. S4A, B). Phenotypic analysis replicated the lymphopenic phenotype observed in *Stk11*^*fl/fl*^*CD122*^*Cre*^ mice, showing significant reductions in NK cell frequencies and absolute counts across multiple tissues (Fig. 2D, E). Subset analysis revealed a developmental blockade at the immature stage, characterized by increased frequencies of CD27^−^CD11b^−^ and CD27^+^CD11b^−^ populations, alongside decreased CD27^+^CD11b^+^ and CD27^−^CD11b^+^ mature subsets (Fig. 2F, G). Surface marker profiling confirmed impaired maturation, with reduced KLRG1 expression and elevated CD117 levels in Lkb1-deficient NK cells compared to the WT control (Fig. 2H). Lastly, we created *Stk11*^*fl/fl*^*Ncr1*^*ERT2-Cre*^ mice, which allowed for tamoxifen-induced acute deletion of Lkb1 specifically in NK cells (Fig. S4C, D). Tamoxifen treatment in adult mice rapidly induced the deletion of Lkb1, leading to a decrease in the number of NK cells and a redistribution of subsets in the spleen and bone marrow (Fig. 2I–L). Collectively, these complementary genetic models provide conclusive evidence that Lkb1 intrinsically regulates NK cell homeostasis through stage-specific control of development and maintenance.

### Lkb1 deficiency induces profound transcriptional reprogramming in NK cells

**Fig. 3:**
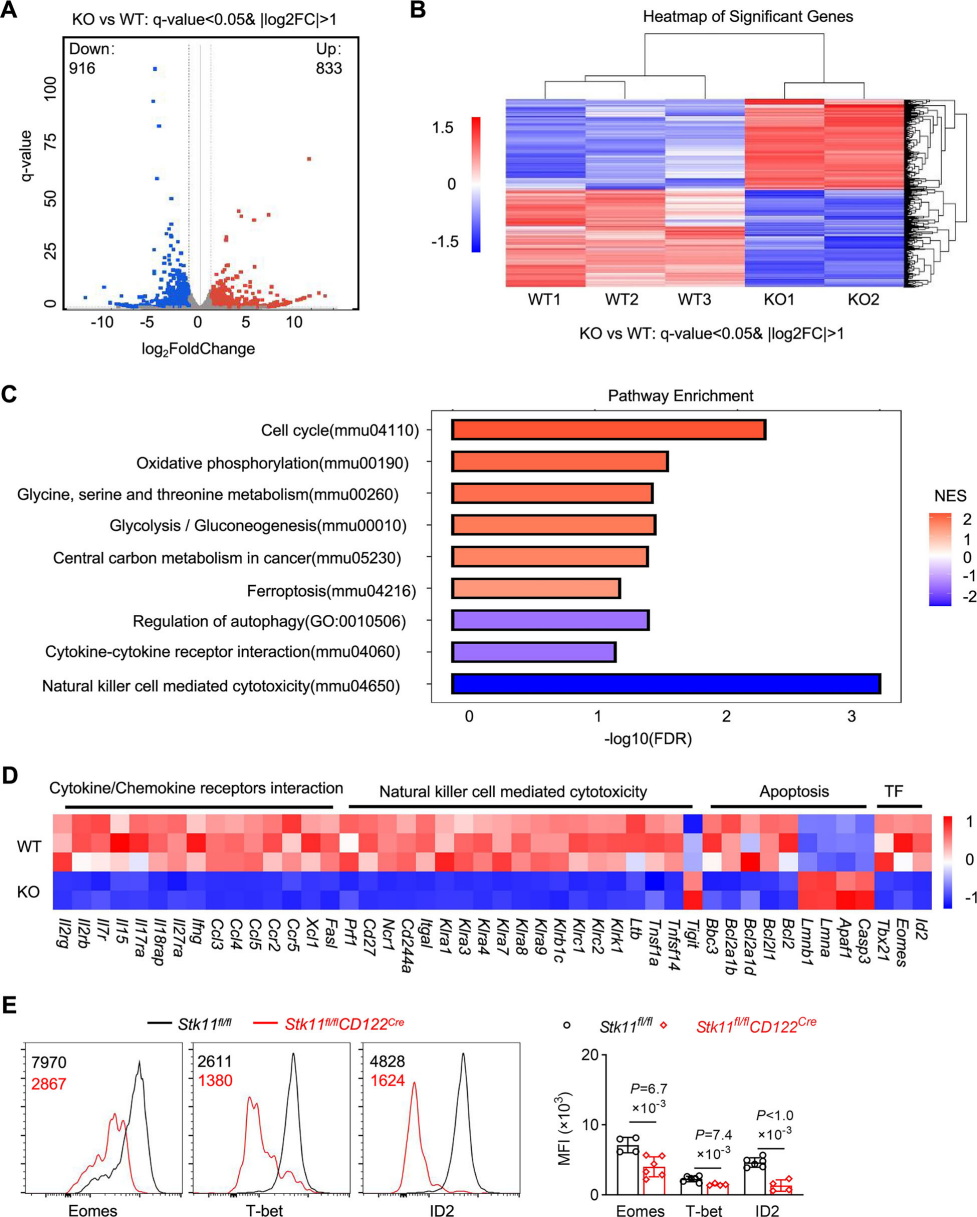
Identification of Lkb1-dependent genes in NK cells. **A** Volcano plot of genes differentially expressed between NK cells from *Stk11*^*fl/fl*^*CD122*^*Cre*^ and *Stk11*^*fl/fl*^ mice (q-value < 0.05, log2FC > 1). **B** Heatmap of genes differentially expressed in Lkb1-depleted NK cells compared to control NK cells. **C** Gene ontology (GO) enrichment analysis of differential transcriptome profiles from Lkb1-depleted versus control NK cells. **D** Heatmap of gene expression related to cytokine/chemokine receptors interaction, natural killer cell-mediated cytotoxicity, apoptosis regulatory molecules, and transcription factors in RNA-seq data. **E** Representative histograms (left) and mean fluorescence intensity (MFI) statistics (right) for Eomes, T-bet, and ID2 in NK cells from *Stk11*^*fl/fl*^ and *Stk11*^*fl/fl*^*CD122*^*Cre*^ mice. Data are presented as mean ± SD. Two-tailed Student’s *t* test (**E**). Data are representative of three (**E**) independent experiments with consistent results.

To evaluate the molecular mechanisms underlying Lkb1-mediated regulation of NK cell development, we conducted an extensive global RNA sequencing (RNA-Seq) analysis comparing sorted NK cells from *Stk11*^*fl/fl*^*CD122*^*Cre*^ (KO) and *Stk11*^*fl/fl*^ (WT) mice. Differential expression analysis identified 1749 significantly altered transcripts (|log2FC| > 1, FDR < 0.05), comprising 833 upregulated and 916 down-regulated genes in Lkb1-deficient NK cells (Fig. 3A, B). Gene ontology (GO) enrichment analysis of these differentially expressed genes revealed striking perturbations in fundamental biological processes, including cell cycle progression, mitochondrial oxidative phosphorylation, ferroptosis signaling, and autophagy regulation (Fig. 3C). Notably, Lkb1 deficiency caused substantial downregulation of critical cytokine receptor genes essential for lymphocyte survival and homeostasis, including *Il2ra*, *Il2rb*, and *Il7r*. Functional analysis demonstrated impaired NK cell effector programming, characterized by reduced expression of *Ifng* alongside elevated *Tigit* levels (Fig. 3D). The apoptotic regulatory network was profoundly disrupted, with significant suppression of anti-apoptotic genes (*Bcl2, Bcl2a1b, Bcl2a1d*) and concomitant upregulation of pro-apoptotic effectors (*Caspase3, Apaf1*). Furthermore, core transcriptional regulators of NK cell identity and function, including *Eomes*, *Tbx21* (T-bet), and *Id*2-, showed markedly reduced expression in Lkb1-deficient NK cells (Fig. 3D). Consistent with these RNA-seq findings, we observed impaired expression of Eomes, T-bet, and ID2 at protein levels by flow cytometric analysis (Fig. 3E). These comprehensive transcriptomic analyses establish that Lkb1 serves as a master transcriptional regulator in NK cells, orchestrating the expression of genes critical for survival, effector function, and developmental progression.

### Lkb1 deficiency disrupts autophagic flux and exacerbates ROS-mediated cytotoxicity in NK cells

**Fig. 4:**
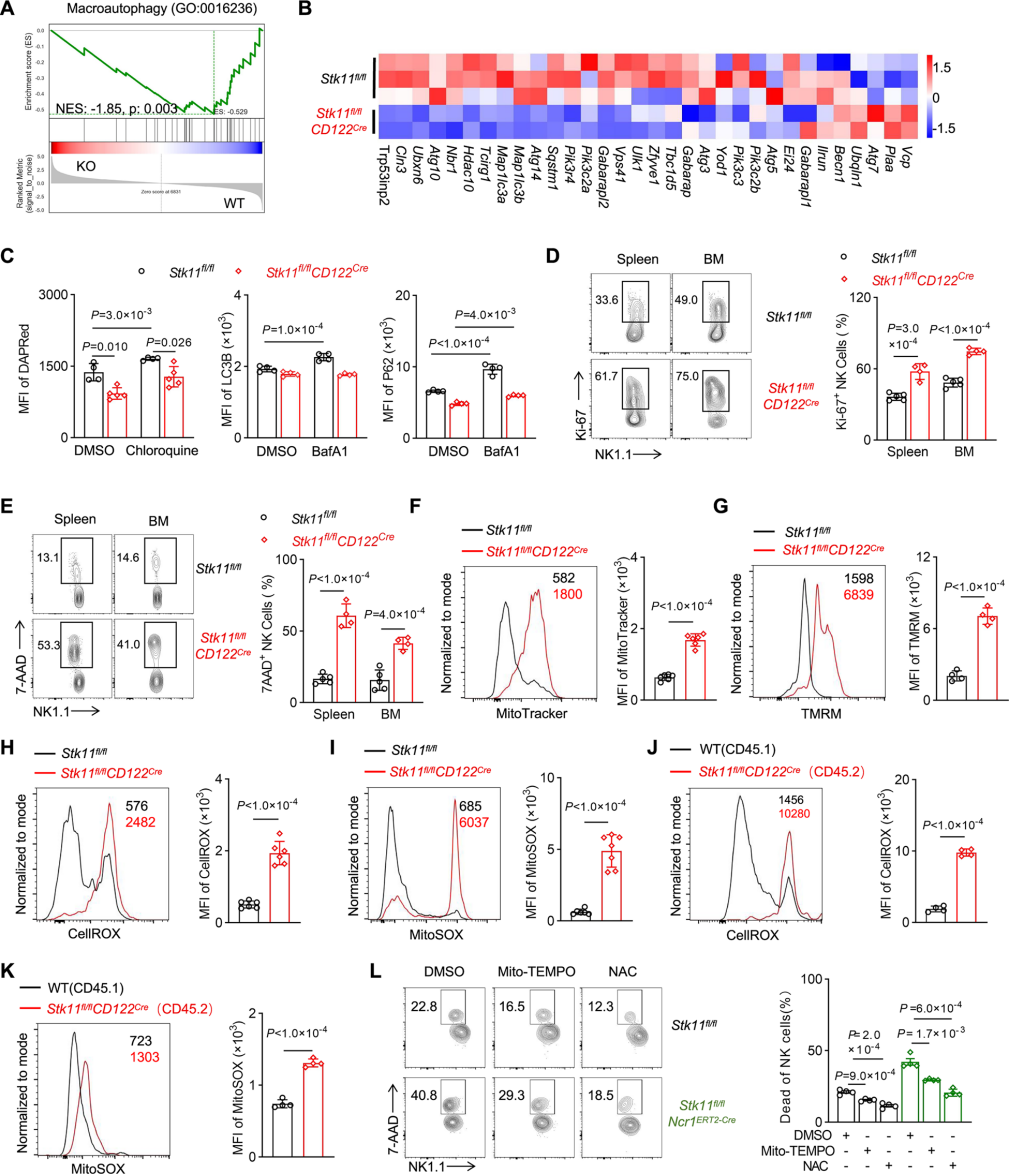
Loss of Lkb1 impairs NK cell autophagy and promotes ROS expression. **A** Gene-set enrichment analysis of RNA-seq data for the enrichment of macro-autophagy signaling-related genes in Lkb1-depleted (KO) NK cells compared with wild-type (WT) control NK cells. **B** Heatmap of gene expression related to macro-autophagy signaling in RNA-seq data. **C** Mean fluorescence intensity (MFI) quantification of DAPRed (left), LC3B (middle) and P62 (right) levels in NK cells from the spleens of *Stk11*^*fl/fl*^ and *Stk11*^*fl/fl*^*CD122*^*Cre*^ mice (*n* = 4–5). **D**, **E** Representative flow cytometry plots (left) and the percentages (right) of Ki-67 (**D**) or 7-AAD (**E**) positive cells in the spleens from *Stk11*^*fl/fl*^ and *Stk11*^*fl/fl*^*CD122*^*Cre*^ mice (*n* = 4–5). **F**–**I** Representative histograms (left) and MFI quantification (right) of MitoTracker (**F**), TMRM (**G**), CellROX (**H**), and MitoSOX (**I**) levels in NK cells from the spleens of *Stk11*^*fl/fl*^ and *Stk11*^*fl/fl*^*CD122*^*Cre*^ mice (*n* = 4–7). **J**, **K** Representative histograms (left) and quantification (right) of CellROX (**J**) and MitoSOX (**K**) levels in CD45.1^+^ or CD45.2^+^ NK cells from the spleens of bone marrow chimeric recipient mice (*n* = 4). **L** Representative flow cytometry graph (left) and the percentage (right) of dead cells in the spleens of *Stk11*^*fl/fl*^ and *Stk11*^*fl/fl*^*Ncr1*^*ERT2-Cre*^ mice induced by tamoxifen in vitro, under conditions with or without NAC (25 mM) or Mito TEMPO (15 μM) treatment for 24 h (*n* = 4). Data are presented as mean ± SD. Two-way ANOVA (**C**, **L**), Two-tailed Student’s *t* test (**D**–**K**). Data are representative of two (**C**–**E**, **L**) or three (**F**–**K**) independent experiments.

Gene-set enrichment analysis (GSEA) revealed a significant reduction in macro-autophagy pathways in Lkb1-deficient NK cells compared to wild-type controls (Fig. 4A). Transcriptomic profiling indicated a coordinated decrease in the expression of core autophagy-related genes (Fig. 4B), which was functionally confirmed by impaired autophagosome formation, as shown by the reduced number of autophagosomes (Figs. 4C, S5A). Further analysis showed that when autophagic flux was inhibited through treatment with bafilomycin A1 (BafA1), the levels of LC3B and p62 significantly increased in WT NK cells, whereas no significant changes in LC3B were observed in Lkb1-deficient NK cells. These findings indicate the critical role of Lkb1 in sustaining normal autophagy processes in NK cells (Figs. 4C, S5B, C). Considering the well-established role of autophagy in maintaining cell survival and tissue homeostasis by degrading damaged organelles, long-lived proteins, and other unnecessary cellular components [30], we systematically assessed the cellular effects of Lkb1 deletion, focusing on changes in proliferation or survival. Phenotypic analysis revealed a paradoxical increase in proliferation alongside higher levels of cell death (7-AAD^+^ cells) (Fig. 4D, E), suggesting that the observed NK cell lymphopenia primarily results from survival issues rather than a defect in proliferation.

In cells with impaired autophagy, damaged mitochondria may accumulate, resulting in abnormally high levels of reactive oxygen species (ROS) [31]. These unregulated ROS levels can have detrimental effects on cell viability. Indeed, we observed an increase in mitochondrial mass and membrane potential in NK cells lacking Lkb1, indicating that the deletion of Lkb1 leads to impaired mitochondrial function (Fig. 4F, G). To evaluate the impact of Lkb1 deficiency on ROS levels in NK cells, we quantified both total and mitochondrial ROS. Our results demonstrated that Lkb1-deficient NK cells exhibited significantly elevated levels of both total and mitochondrial ROS compared to their wild-type counterparts (Fig. 4H, I). These observations suggest that the absence of Lkb1 disrupts mitochondrial function, promoting the production of heightened ROS levels. These findings were consistently replicated across multiple genetic models, including mixed chimeras, *Stk11*^*fl/fl*^*Ncr1*^*iCre*^ mice, and *Stk11*^*fl/fl*^*Ncr1*^*ERT2-cre*^ mice (Figs. 4J, K, S6A–H), confirming that these effects are cell-intrinsic.

To further investigate whether the elevated ROS levels in Lkb1-deficient NK cells contributed to their reduced survival, we administered treatment to the cells using the ROS scavenger N-acetyl-L-cysteine (NAC) or the mitochondrial-specific ROS scavenger Mito-TEMPO. Remarkably, the scavenging of ROS substantially alleviated the survival deficit observed in Lkb1-deficient NK cells (Fig. 4L), suggesting that the excessive accumulation of ROS is associated with the impaired survival of these cells. These data collectively demonstrate that Lkb1 serves as a critical regulator of NK cell homeostasis by maintaining autophagic flux, preventing mitochondrial dysfunction, and reducing ROS-induced cytotoxicity.

### Lkb1 deficiency coordinately regulates apoptotic and iron ion-dependent cell death pathways in NK cells

**Fig. 5:**
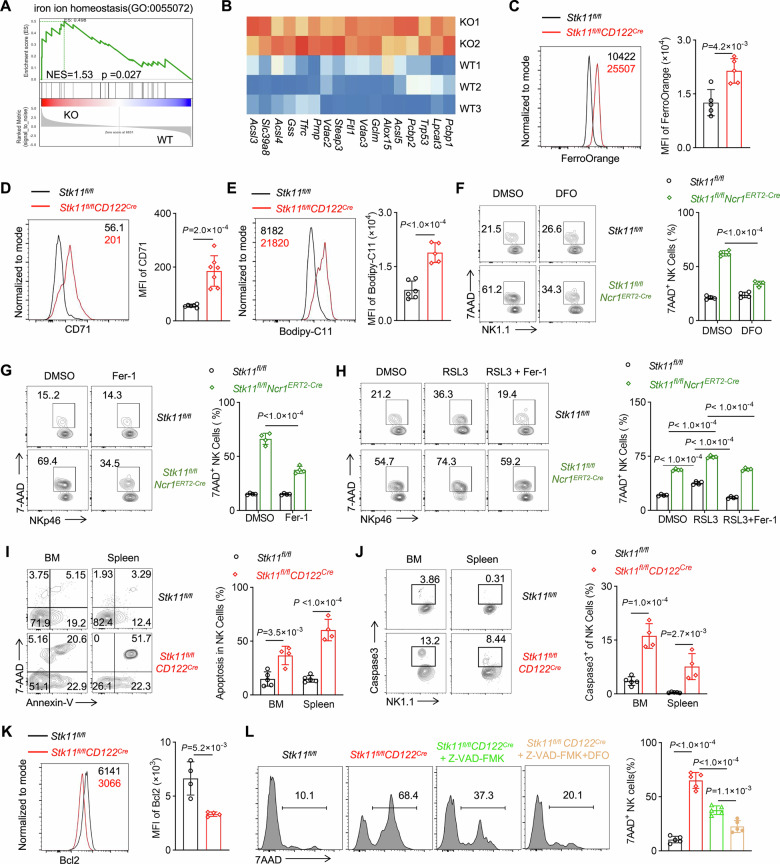
Loss of Lkb1 in NK cells induces apoptosis and iron ion-dependent cell death. **A** Gene-set enrichment analysis of RNA-seq data for the enrichment of iron ion homeostasis-related genes in Lkb1-depleted (KO) NK cells compared with WT control NK (WT) cells. **B** Heatmap of the gene expression involved in iron ion homeostasis in RNA-seq data. **C**–**E** Representative histograms (left) and MFI quantification (right) of FerroOrange (**C**), CD71 (**D**), and Bodipy-C11 (**E**) levels in NK cells from spleens of *Stk11*^*fl/fl*^ and *Stk11*^*fl/fl*^*CD122*^*Cre*^ mice (*n* = 4–7). **F** Representative flow cytometry plots (left) and percentage (right) of 7-AAD-positive NK cells from tamoxifen-induced *Stk11*^*fl/fl*^ and *Stk11*^*fl/fl*^*Ncr1*^*ERT2-Cre*^ mice treated with or without DFO for 24 h (*n* = 4–5). **G** Representative flow cytometry plots (left) and the percentages (right) of 7-AAD-positive NK cells from tamoxifen-induced *Stk11*^*fl/fl*^ and *Stk11*^*fl/fl*^*Ncr1*^*ERT2-Cre*^ mice treated with or without Fer-1 for 24 h (*n* = 4). **H** Representative flow cytometry plots (left) and the percentages (right) of 7-AAD-positive NK cells in the spleens of tamoxifen-induced *Stk11*^*fl/fl*^ and *Stk11*^*fl/fl*^*Ncr1*^*ERT2-Cre*^ mice treated with RSL3 (1 μM), or RSL3 (1 μM) combined with Fer-1 (1 μM) for 24 h (*n* = 4). **I** Representative flow cytometry plots (left) and percentage (right) of Annexin V-positive NK cells in BM and spleen from *Stk11*^*fl/fl*^ and *Stk11*^*fl/fl*^*CD122*^*Cre*^ mice (*n* = 4–5). **J** Representative flow cytometry plots (left) and percentage (right) of caspase 3-positive NK cells in BM and spleen from *Stk11*^*fl/fl*^ and *Stk11*^*fl/fl*^*CD122*^*Cre*^ mice (*n* = 4–5). **K** Representative histograms (left) and MFI quantification (right) of Bcl2 levels in NK cells from spleens of *Stk11*^*fl/fl*^ and *Stk11*^*fl/fl*^*CD122*^*Cre*^ mice (*n* = 4). **L** Representative histograms (left) and percentage (right) of 7-AAD-positive NK cells from *Stk11*^*fl/fl*^ and *Stk11*^*fl/fl*^*CD122*^*Cre*^ mice treated with Z-VAD-FMK, or Z-VAD-FMK combined with DFO (*n* = 5). Data are presented as mean ± SD. Two-tailed Student’s *t* test (**C**–**E**, **I**–**K**), Two-way ANOVA (**F**–**H**), One-way ANOVA (**L**). Data are representative of three independent experiments with consistent results.

To elucidate the mechanisms underlying Lkb1-mediated cell death regulation, we conducted comprehensive analyses of cell death pathways in NK cells. Gene Set Enrichment Analysis (GSEA) revealed significant enrichment of iron homeostasis-related gene signatures in Lkb1-deficient NK cells (Fig. 5A, B), prompting further investigation into iron metabolism. Quantitative analysis revealed substantial iron accumulation in Lkb1-deficient NK cells compared to wild-type controls (Fig. 5C), suggesting impaired iron regulatory mechanisms. The iron uptake receptor CD71 (*Tfrc*), a known mediator of ferroptosis [32, 33], was markedly upregulated in Lkb1-deficient NK cells at both transcriptional and protein levels (Fig. 5B, D). This aberrant iron metabolism was accompanied by elevated lipid peroxidation, as evidenced by increased lipid ROS accumulation (Fig. 5E). These findings were consistently reproduced in *Stk11*^*fl/fl*^*Ncr1*^*iCre*^, confirming cell-intrinsic dysregulation of iron homeostasis (Fig. S7A, B). To further elucidate the impact of accumulated iron ions on the survival of Lkb1-deficient NK cells, we treated NK cells derived from *Stk11*^*fl/fl*^ and *Stk11*^*fl/fl*^*Ncr1*^*ERT2-Cre*^ mice with the iron chelator deferoxamine (DFO). The results revealed that the removal of excess iron ions significantly restored the viability of Lkb1-deficient NK cells (Fig. 5F). To verify the specific contribution of ferroptosis to Lkb1-deficient NK cell survival, we administered the classical ferroptosis inhibitor Ferrostatin-1 (Fer-1) to NK cells. The results showed that Fer-1 could markedly reduce the cell death caused by Lkb1 deficiency (Fig. 5G). Furthermore, we found that the absence of Lkb1 disrupted the normal expression of several iron ferroptosis-related proteins, including MDA, GPX4 and ACSL4 (Fig. S7C–E). When NK cells were exposed to the ferroptosis inducer RSL3, Lkb1-deficient NK cells exhibited significantly lower viability compared to WT NK cells. The additional administration of Fer-1 could substantially reverse this effect (Fig. 5H). Collectively, these findings indicate that Lkb1 deficiency disrupts iron homeostasis, leading to lipid peroxidation and ferroptotic cell death. This mechanistic insight establishes Lkb1 as a critical regulator of iron metabolism and oxidative stress response in NK cells.

Complementary to the observed ferroptosis phenotype, transcriptomic analysis revealed significant dysregulation of apoptosis-related genes in Lkb1-deficient NK cells (Fig. 3D). Flow cytometric quantification using Annexin V/7AAD staining confirmed substantially increased apoptotic rates in Lkb1-deficient NK populations compared to wild-type controls (Fig. 5I). Molecular characterization demonstrated a pronounced apoptotic signature, characterized by elevated caspase-3 expression and concomitant suppression of Bcl-2 anti-apoptotic signaling in *Stk11*^*fl/fl*^*CD122*^*Cre*^ mice relative to controls (Fig. 5J, K). Moreover, pharmacological intervention studies provided mechanistic validation: the pan-caspase inhibitor Z-VAD-FMK partially rescued cell viability, while co-treatment with Z-VAD-FMK and DFO exhibited additive protective effects (Fig. 5L). These findings establish that Lkb1 serves as a master regulator of NK cell survival through dual mechanisms: maintaining mitochondrial integrity to prevent intrinsic apoptosis, and preserving iron homeostasis to avert ferroptotic cell death. The synergistic rescue by DFO and Z-VAD-FMK suggests these pathways operate independently yet contribute collectively to NK cell depletion in Lkb1 deficiency.

### The function of Lkb1 in NK cells could not be rescued by pharmacologic AMPK activation or mTORC1 inhibition

**Fig. 6:**
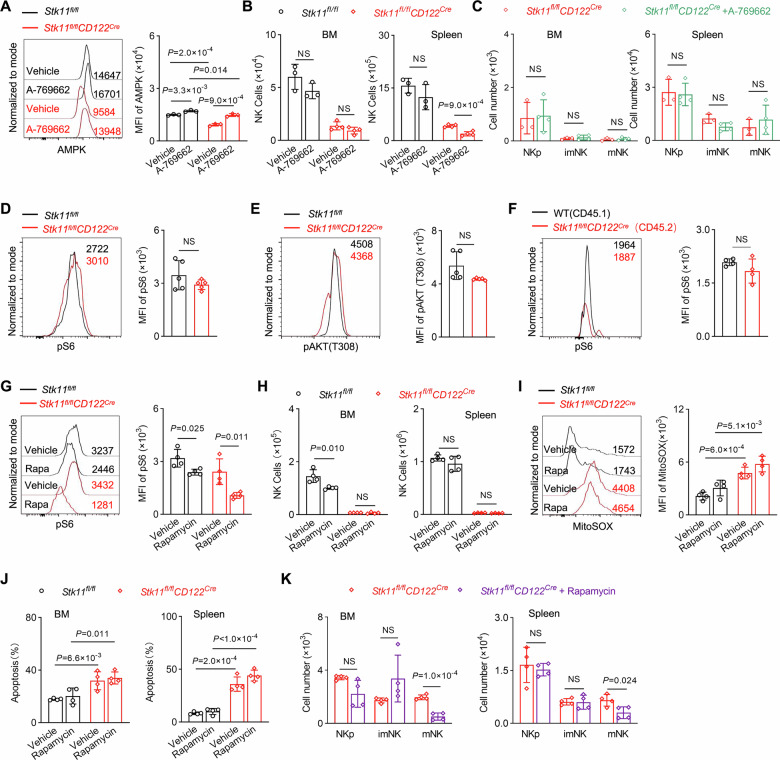
Pharmacologic AMPK activation or mTORC1 inhibition could not restore the homeostatic and developmental defects of NK cells caused by Lkb1 deficiency. **A** Representative histograms (left) and quantification (right) of AMPK expression in NK cells from the spleens of *Stk11*^*fl/fl*^ and *Stk11*^*fl/fl*^*CD122*^*Cre*^ mice, treated with or without A-769662 (*n* = 3). **B** Quantification of the absolute number of NK cells in the bone marrow (BM) and spleen from *Stk11*^*fl/fl*^ and *Stk11*^*fl/fl*^*CD122*^*Cre*^ mice, treated with or without A-769662 (*n* = 4). **C** Quantification of the absolute number of NKp, imNK, and mNK cells in the BM and spleen from *Stk11*^*fl/fl*^ and *Stk11*^*fl/fl*^*CD122*^*Cre*^ mice, treated with or without A-769662 (*n* = 3–4). **D**, **E** Representative histograms (left) and MFI quantification (right) of pS6 (**D**) and pAKT (T308) (**E**) in NK cells from the spleens of *Stk11*^*fl/fl*^ and *Stk11*^*fl/fl*^*CD122*^*Cre*^ mice (*n* = 5). **F** Representative histograms (left) and MFI quantification (right) of pS6 levels in CD45.1^+^ or CD45.2^+^ NK cells from the spleens of bone marrow chimeric recipient mice (*n* = 4). **G** Representative histograms (left) and MFI quantification (right) of pS6 expression in NK cells from the spleens of *Stk11*^*fl/fl*^ and *Stk11*^*fl/fl*^*CD122*^*Cre*^ mice, treated with or without Rapamycin (*n* = 4). **H** Quantification of the absolute number of NK cells in the BM (left) and spleen (right) from *Stk11*^*fl/fl*^ and *Stk11*^*fl/fl*^*CD122*^*Cre*^ mice, treated with or without Rapamycin (*n* = 4). **I** Representative histograms (left) and MFI quantification (right) of MitoSOX levels in NK cells from the spleens of *Stk11*^*fl/fl*^ and *Stk11*^*fl/fl*^*CD122*^*Cre*^ mice, treated with or without Rapamycin (*n* = 4). **J** The percentage of Annexin-V^+^ NK cells from the BM (left) and spleen (right) of *Stk11*^*fl/fl*^ and *Stk11*^*fl/fl*^*CD122*^*Cre*^ mice, treated with or without Rapamycin (*n* = 4). **K** The absolute numbers of NKp, imNK, and mNK cells in the BM (left) and spleen (right) of *Stk11*^*fl/fl*^ and *Stk11*^*fl/fl*^*CD122*^*Cre*^ mice, treated with or without Rapamycin (*n* = 4). Data are presented as mean ± SD. Two-way ANOVA (**A**, **G**, **I**, **J**), Two-tailed Student’s *t* test (**B**–**F**, **H**, **K**); NS, not significant. Data are compiled from three (**A**–**E**, **G**–**K**) or two (**F**) independent experiments.

AMPK and mTORC1 are both recognized as key signaling pathways involved in the energy stress response regulated by Lkb1 [18, 34]. We also found that the expression of AMPK was decreased in Lkb1-deficient NK cells compared to the controls (Fig. 6A). To determine the functional contribution of AMPK signaling, we employed a pharmacological rescue approach using the specific AMPK activator A-769662 in *Stk11*^*fl/fl*^*CD122*^*Cre*^ mice. The compound demonstrated expected target engagement, evidenced by AMPKα1/α2-dependent phosphorylation of ACC at Ser79 [35], confirming its biological activity independent of Lkb1 status (Fig. 6A). Despite effectively restoring AMPK activity, A-769662 treatment failed to rescue either the survival defects or the abnormal subset distribution of Lkb1-deficient NK cells (Figs. 6B, C, S8A, B). These data suggest that while Lkb1 deficiency impairs AMPK activation, the essential role of Lkb1 in NK cell maintenance operates through alternative pathways.

While Lkb1 is well-established as a negative regulator of mTOR signaling across multiple cell types, with its deficiency leading to constitutive mTOR activation in cancer cells and T cells [29, 34, 36], we observed distinct regulatory patterns in NK cells. Strikingly, both steady-state analysis and mixed bone marrow chimera experiments revealed normal mTORC activity in Lkb1-deficient NK cells, evidenced by unaltered p-S6 level, p-AKT308 level, p-AKT473 level and p-4E-BP1 level (Figs. 6D–F, S8C, D). Although rapamycin treatment effectively suppressed the phosphorylation of S6 (Fig. 6G), it failed to alleviate any of the pathological phenotypes observed in *Stk11*^*fl/fl*^*CD122*^*Cre*^ mice, including impaired cellularity, elevated ROS levels, increased apoptosis, and developmental defects (Figs. 6H–K, S8E–G). In addition, we generated a new genetic animal with haploinsufficiency of *Rptor* in *Stk11*^*fl/fl*^*CD122*^*Cre*^ mice, which allowed partly defect of mTORC1 activity within Lkb1-deficient NK cells (*Stk11*^*fl/fl*^*Rptor*^*fl/+*^*CD122*^*Cre*^). The results showed that the reduction of mTORC1 signaling could not rescue the homeostatic and developmental defects of NK cells caused by Lkb1 deficiency (Fig. S9A–D). These comprehensive research results indicate that, unlike the established roles in other cell types, the imbalance in NK cell homeostasis caused by Lkb1 deficiency cannot be restored by activating AMPK or inhibiting the mTORC1 pathway.

### Deletion of Lkb1 impairs NK cell function and compromises NK-mediated immunosurveillance

**Fig. 7:**
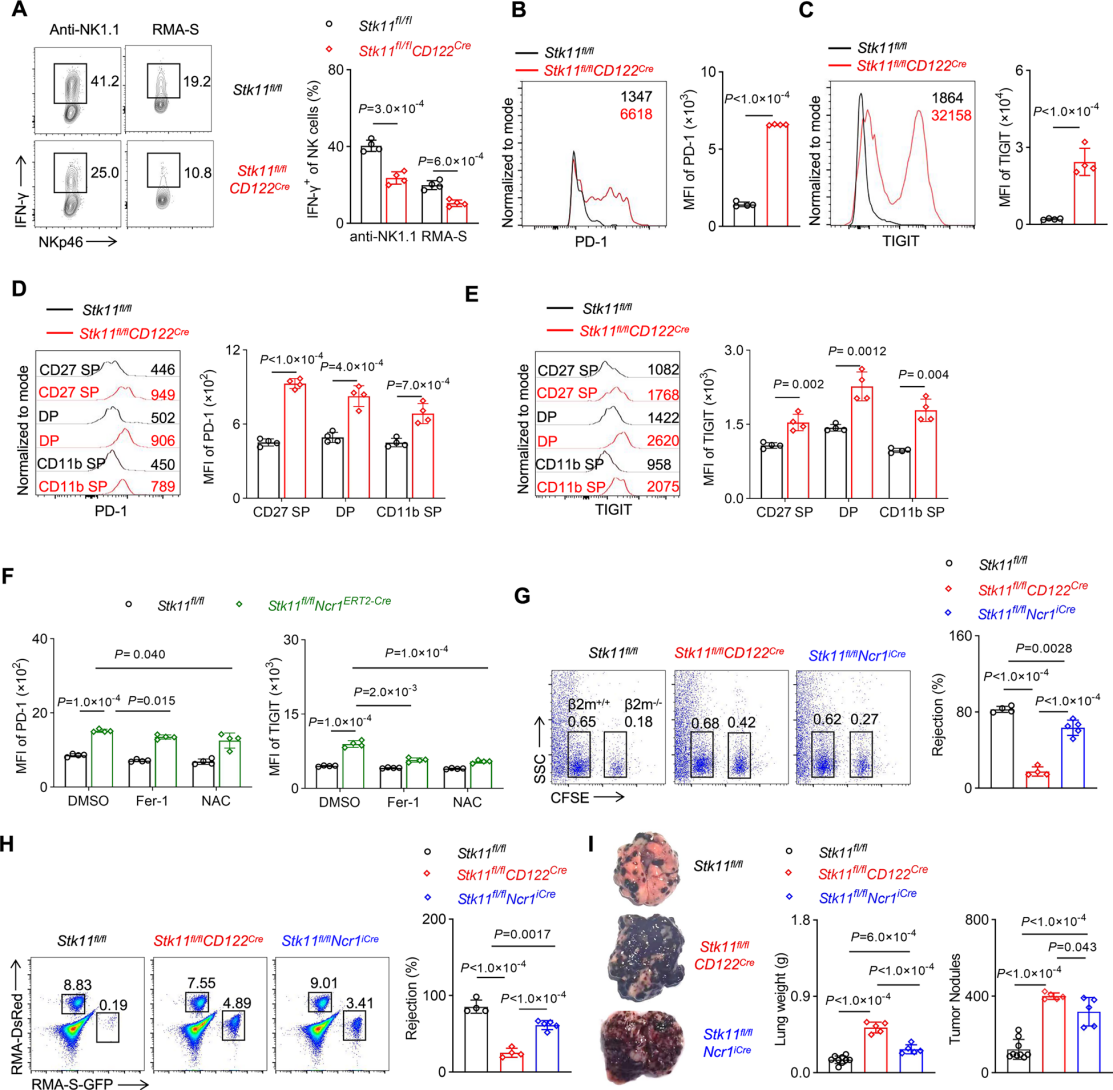
Lkb1 controls NK cell function. **A** Representative flow cytometry plots (left) and percentage (right) of IFN-γ^+^ NK cells from *Stk11*^*fl/fl*^ and *Stk11*^*fl/fl*^*CD122*^*Cre*^ mice stimulated with anti-NK1.1 or RMA-S (*n* = 4). **B**, **C** Representative plots (left) and MFI quantification (right) of PD-1 (**B**) and TIGIT (**C**) in spleen NK cells from *Stk11*^*fl/fl*^ and *Stk11*^*fl/fl*^*CD122*^*Cre*^ mice (*n* = 4). **D** Representative histograms (left) and MFI quantification (right) of PD-1 (**D**), and TIGIT (**E**) levels of different subsets in NK cells from the spleens of *Stk11*^*fl/fl*^ and *Stk11*^*fl/fl*^*CD122*^*Cre*^ mice (*n* = 4). **F** MFI quantification of PD-1 (left), and TIGIT (right) levels in spleens of Stk11^fl/fl^ and *Stk11*^*fl/fl*^*Ncr1*^*ERT2-Cre*^ mice treated with NAC or Fer-1 for 24 h (*n* = 4). **G** Representative flow cytometry plots (left) and percentage (right) of rejection rates for *β2m*^−/−^ splenocytes obtained from the spleens of the indicated recipient mice after injection with an equal number of *β2m*^+/+^ or *β2m*^−/−^ splenocytes labeled with various concentrations of CFSE (*n* = 4–5). **H** Representative plots (left) and percentages (right) of rejection rates for RMA-S cells in the peritoneal cavity of the indicated recipient mice after intraperitoneal injection with a mixture containing GFP-expressing RMA-S cells sensitive to NK cell killing and DsRed-expressing RMA cells resistant to NK cell killing (*n* = 4–5). **I** Representative photographs (left), number of tumor nodules (middle), and lung weights (right) in the indicated mice (*n* = 5–9). Data are presented as mean ± SD. Two-tailed Student’s *t* test (**A**–**E**), Two-way ANOVA (**F**), One-way ANOVA (**G**–**I**). Data are compiled from 3 (**A**–**C**) or ≥2 (**D**–**I**) independent experiments.

Transcriptomic analysis revealed that Lkb1-deficient NK cells displayed a dual molecular signature, characterized by the downregulation of cytotoxic effector genes and the upregulation of exhaustion markers (Fig. 3D). Functional assays further substantiated these findings, revealing significantly impaired IFN-γ production in Lkb1-deficient NK cells following stimulation with anti-NK1.1 or RMA-S, in contrast to their wild-type counterparts (Fig. 7A). Furthermore, Lkb1-deficient NK cells exhibited increased surface expression of the exhaustion markers PD-1 and TIGIT (Fig. 7B, C). This increased expression of exhaustion molecules was uniformly observed across all NK cell subsets, indicating that it was not attributable to shifts in subpopulation proportions (Fig. 7D, E). To investigate whether oxidative damage resulting from Lkb1 deficiency contributes to NK cell dysfunction, we treated Lkb1-deficient NK cells with Fer-1 or NAC. The results confirmed that reducing oxidative stress significantly suppressed the expression of PD-1 and TIGIT in Lkb1-deficient NK cells (Figs. 7F, S10A). Furthermore, Fer-1 or NAC treatment enhanced the production of IFN-γ and CD107a in Lkb1-deficient NK cells (Fig. S10B, C), indicating that the impaired functionality caused by Lkb1 deficiency in NK cells can be partially reversed by antioxidants.

NK cells are crucial in the rejection of allogeneic cells and tumors. To further investigate the impact of Lkb1 on NK cell function, we conducted in vivo experiments. Initially, we assessed the ability of NK cells to mediate “missing-self” rejection. Our findings showed that NK cells in wild-type mice efficiently eradicated MHC-I-deficient splenocytes, whereas Lkb1-deficient mice exhibited a significant reduction in NK cell-mediated cytotoxicity (Fig. 7G). Additionally, Lkb1-deficient NK cells showed substantial deficiencies in their capacity to eliminate tumor cells, as evidenced by the clearance of the NK-sensitive tumor cell line RMA-S (Fig. 7H). Moreover, in the melanoma lung metastasis model, Lkb1-deficient mice displayed significantly higher lung weight and tumor colony numbers compared to control mice (Fig. 7I). Comparative analysis of *Stk11*^*fl/fl*^*CD122*^*Cre*^ and *Stk11*^*fl/fl*^*Ncr1*^*iCre*^ mice demonstrated that Lkb1 deletion at the NKp stage resulted in more severe functional impairment, indicating that the loss of Lkb1 at the NKp stage has a greater impact on NK cell function and tumor surveillance. Collectively, these findings establish Lkb1 as a critical regulator of NK cell function, maintaining immunosurveillance capacity by preventing exhaustion and preserving cytotoxic potential.

## Discussion

Despite the recent focus on the pathways that trigger metabolic reprogramming in NK cells [37, 38], the mechanisms underlying the negative regulation of NK cell metabolism remain poorly understood. In this study, we elucidated that Lkb1 signaling is critical for maintaining NK cell homeostasis through multiple interconnected pathways. These include preserving cellular quiescence to prevent over-activation, keeping mitochondrial integrity and autophagic processes, and regulating iron metabolism to prevent the accumulation of lipid ROS. The functional consequences of Lkb1 deficiency are significant, encompassing heightened expression of exhaustion markers, impaired cell survival, and, ultimately, compromised antitumor surveillance capabilities. Thus, Lkb1 functions as a central signaling node that establishes metabolic and immune equilibrium in NK cells, thereby promoting immune homeostasis and enhancing antitumor immune responses.

NK cells represent a critical component of innate immunity with well-documented roles in antiviral and antitumor immunity [39]. Their functional competence depends on both quantitative maintenance of NK cell subsets and qualitative regulation of surface receptor expression, which collectively determine immune surveillance efficacy and subsequent adaptive immune responses. Our study elucidated the pivotal role of Lkb1 in NK cell development through stage-specific analyses. Lkb1 deficiency disrupts NK cell maturation, leading to severe systemic lymphopenia across multiple organs. This phenotype was characterized by the accumulation of immature NK cells and a concurrent reduction in mature subsets, indicating a developmental blockade after Lkb1 deficiency. Notably, when comparing the two mouse models, *Stk11*^*fl/fl*^*CD122*^*Cre*^ mice (with Lkb1 deletion at the NKp stage) exhibited more severe NK cell defects compared to *Stk11*^*fl/fl*^*Ncr1*^*iCre*^ mice (with Lkb1 deletion at the iNK stage). These findings establish that Lkb1 serves as a critical regulator of NK cell homeostasis throughout the entire process of NK cell development. Its significance is particularly pronounced during the early stages of NK cell commitment.

NK cell numbers are typically determined by the dynamic equilibrium between cellular proliferation and death. Our research has revealed that although the cell proliferation rate in Lkb1-deficient mice is higher than that in the wild-type control group, the number of NK cells in them has significantly decreased. This apparent contradiction was resolved by demonstrating that Lkb1 deficiency leads to substantially elevated apoptosis rates, which outweigh the enhanced proliferative capacity. These findings establish that Lkb1 primarily maintains NK cell homeostasis by promoting cell survival, suggesting its essential role in protecting NK cells from programmed cell death. The observed proliferative increase in Lkb1-deficient NK cells may represent a compensatory mechanism attempting to counterbalance the excessive cell loss, further underscoring the crucial survival function of Lkb1 in NK cell biology.

As a vital regulatory molecule, aberrant Lkb1 expression can elicit changes in multiple genes and signaling pathways [40, 41]. RNA-seq profiling of Lkb1-deficient NK progenitor cells demonstrated genome-wide transcriptional alterations, with GO analysis highlighting significant enrichment in four critical biological processes: cell cycle regulation, oxidative phosphorylation, ferroptosis, and autophagy control. The observed autophagy dysregulation is particularly noteworthy given its fundamental role in cellular quality control through autophagosome-mediated degradation of damaged organelles and proteins [42]. Our data demonstrate that Lkb1 deficiency leads to both downregulation of autophagy-related genes and impaired autophagic flux, mirroring the phenotypic consequences seen in NK cells lacking core autophagy components VPS34 or ATG5 [43, 44]. These findings position Lkb1 upstream of the autophagy machinery in NK cells, suggesting it functions as a critical node linking metabolic sensing to cellular housekeeping mechanisms. Emerging evidence highlights the critical interplay between autophagy and ROS regulation, where mitophagy serves as a key mechanism for eliminating damaged mitochondria and degrading ROS-generating enzymes to control ROS production. In Lkb1-deficient NK cells, we observed heightened levels of both total and mitochondrial ROS, accompanied by an increase in mitochondrial mass and membrane potential. Notably, ROS scavengers markedly reduced NK cell death, implying ROS overload as a major contributor to Lkb1 deficiency-induced cell death. The observed iron overload likely exacerbates oxidative stress through Fenton chemistry while simultaneously triggering iron-dependent cell death pathways. ROS scavenger and ferroptosis inhibitor could significantly reduce cell death and restore the functional impairment caused by Lkb1 deficiency. The convergence of Lkb1 and autophagy pathways in maintaining NK cell homeostasis and function raises important questions about their potential crosstalk with other identified processes (oxidative phosphorylation and ferroptosis), warranting further investigation into the hierarchical relationships among these pathways in NK cell biology.

Although AMPK and mTORC1 are well-established downstream effectors of Lkb1 in energy stress responses, we observed that while AMPK was indeed inactivated in Lkb1-deficient NK cells, pharmacological activation of AMPK failed to rescue the survival defects or population dynamics. Similarly, mTORC1 activity remained unchanged in Lkb1-deficient NK cells under basal conditions, and mTOR inhibition with rapamycin, despite effectively suppressing S6 phosphorylation, showed no therapeutic benefit in terms of NK cell numbers, ROS accumulation, apoptosis rates, or developmental defects. This finding is particularly significant as it challenges the prevailing paradigm of Lkb1 function and points to the existence of novel, cell-type-specific effector mechanisms. Future studies should focus on identifying these alternative pathways, particularly those involved in redox regulation, to fully elucidate the molecular basis of Lkb1-mediated NK cell survival and homeostasis.

Although we have reached these conclusions, there are still numerous limitations in this study that require further exploration. The use of mouse models may not fully replicate human pathophysiology. The specific role of Lkb1 in the development and functional maturation of human NK cells requires further verification. Considering claims about manipulating Lkb1 (a tumor suppressor), caution is needed. Targeting Lkb1 pathways may have benefits, but risks exist, as altering its function may promote oncogenic processes. It is wise to explore alternatives focusing on its downstream effectors, like redox and iron homeostasis nodes. Targeting these can disrupt the tumor-promoting environment or boost therapy efficacy without harming Lkb1 tumor-suppressive role.

## Materials and methods

### Mice

*Stk11*^*flox/flox*^ mice, *Rptor*^*flox/flox*^ mice, CD45.1 mice, and *β2m*-deficient mice were purchased from Jackson Laboratory. *CD122*^*Cre/+*^ mice were generated in Zhongjun Dong lab. *Ncr1-iCre*^*Tg*^ mice and *Ncr1*^*ERT2-Cre*^ mice were purchased from Shanghai Model Organisms Center, Inc. Sex- and age-matched female and male mice were used in our experiments. To induce *Stk11* deletion, *Stk11*^*fl/fl*^*Ncr1*^*ERT2-Cre*^ mice were treated with tamoxifen (100 mg/kg in corn oil) every other day for five doses. Control mice received corn oil only. Body weight was monitored to assess toxicity; a mild, transient weight loss (<10%) occurred after the first injection but rapidly recovered, with no significant long-term differences between groups. All mice were C57BL/6 background and maintained in specific pathogen-free animal facilities of Jinan University.

### Cell lines

B16F10 cells were cultured in DMEM (Hyclone) supplemented with 10% FBS (Gibco). RMA-S cells and RMA cells were cultured in RPMI-1640 (Hyclone) supplemented with 10% FBS. Primary NK cells were cultured in complete medium (RPMI 1640 basal medium supplemented with 10% FBS, 50 μM 2-mercaptoethanol and 1000 U/mL IL-2). To induce Lkb1 knockdown, 1 μg/mL tamoxifen treatment was added during culture for a total of 6 days. After the knockdown induction was completed, for mechanistic studies, the cells were treated with inhibitors targeting different pathways for 24 h, including: NAC (25 mM), DFO (100 μM), (1S,3R)-RSL3 (1 μM), Mito TEMPO (10 μM) or Ferrostatin-1 (1 μM). All chemicals were purchased from MedChemExpress (MCE). All cells were maintained at 37 °C incubator containing 5% CO_2_.

### Flow cytometry

Flow cytometry was performed on BD FACSVerse™ (three-laser flow cytometry analyzer, BD Biosciences) or CYTEK NORTHERN LIGHTS (a 3 Laser 16V-14B-8R, CYTEK). Monoclonal antibodies against mouse CD3 (145-2C11), CD122 (TM-b1), CD71 (R17217), CD117 (2B8), CD127 (A7R34), Bcl-2 (10C4), KLRG1 (2F1), Ly49D (4E5), Ly49H (3D10), Ly49G2 (4D11), NK1.1 (PK136), NKp46 (29A1.4), NKG2A (16a11), Eomes (Dan11mag), T-bet (4B10), ID2 (ILCID2), PD-1 (J43), TIGIT (GIGD7), CD49b (DX5), AMPK (2B7) and isotype controls were purchased from eBioscience (San Diego, CA). Monoclonal antibodies against mouse CD11b (M1/70), CD27 (LG.3A10), CD45.1 (A20), CD45.2 (Ly-5.2), Ki-67 (SolA15), Annexin V (Cat#640941), IFN-γ (XMG1.2) and Ly49A (YE1/48.10.6) were purchased from Biolegend (San Diego, CA). Monoclonal antibodies against mouse Caspase3 (C92-605), 7AAD (Cat#559925) and Ly49C/I (5E6) were purchased from BD Biosciences (Mississauga, Ontario, Canada). Anti-phospho-AKT (Thr308) (D25E6) and Anti-phospho-S6 (D57.2.2E) were obtained from Cell Signaling Technology (Beverly, MA). Dead cells were excluded from analysis by using LIVE/DEAD fixable Aqua dead cell stain kit (Cat#L34965, Invitrogen) according to the manufacturer’s instructions. MitoSOX Red (Cat #M36008, Invitrogen) and CellROX Deep Red (Cat #C10422, Invitrogen) were used to detect the mitochondrial and intracellular total ROS in NK cells, respectively. Tetramethylrhodamine (TMRM) (Cat#T668, Invitrogen) and MitoTracker® Mitochondrion-Selective Probes (Cat#M22426, Invitrogen) were used to detect mitochondrial potential and mitochondrial mass in NK cells. BODIPY 581/591 C11 (Fisher Scientific, D3861) was used to detect lipid peroxidation. Anti-LC3B, P62 were used to detect the protein levels of LC3B and P62 in NK cells, respectively. Detection of autophagic flow was achieved by flow cytometry analysis of changes in protein levels of LC3B and P62 before and after 6 h of Bafilomycin A1 (BafA1) treatment. FerroOrange (Cat #F374, Dojindo) was used to detect the intracellular Fe^2+^ levels in NK cells. For analysis of surface markers, cells were stained in PBS containing 2% (w/v) BSA at room temperature. Intracellular staining was performed using Foxp3/transcription factor staining buffer kit (Cat #00-5523-00, eBioscience) according to the manufacturer’s instructions. Flow cytometry data were analyzed using Flowjo software.

### RNA isolation and RT-qPCR

Total RNA was extracted from *Stk11* ^*fl/fl*^ (WT) and *Stk11*^*fl/fl*^*CD122*^*Cre*^ (KO) splenic NK cells, and reverse transcription was performed with Takara Reverse Transcription Kit. The primers for the mouse gene were as follows: *Stk11*-F: GCCTGGAATACCTACACAGCCA; *Stk11*-R: GCAGGTGTCATCCACAGCGAAA. The primers for the endogenous gene *Gapdh* were: *Gapdh*-F: CCAGCTTAGGTTCATCAGGT, and *Gapdh*-R: TTGATGGCAACAATCTCCAC. RT-qPCR was performed using a TB Green Premix Ex Taq kit (TaKaRa Biotechnology, Shiga, Japan) and a BioRad CFX Connect cycler according to the manufacturer’s instructions. The RT-qPCR procedure was as follows: two-step amplification (35 cycles): pre-denaturation (95 °C, 10 min), denaturation (95 °C, 15 s) and annealing/extension (60 °C, 1 min).

### Bone marrow reconstitution

Wild-type mice (CD45.1^+^) and *Stk11*^*fl/fl*^*CD122*^*Cre*^ (CD45.2^+^) mice were treated with 300 μL 10 mg/mL 5-FU. The bone marrow cells were obtained by flushing the femurs and tibias after 4 days. For competitive transfers, BM cells from CD45.1^+^ WT and *Stk11*^*fl/fl*^*CD122*^*Cre*^ mice were mixed in a 1:1 ratio, and then a total of 1 × 10^7^ mixed BM cells were injected i.v. into recipient mice (CD45.1^+^ CD45.2^+^), which was sub-lethally irradiated with 6 Gy. After 8 weeks, the reconstruction of recipients was assessed by flow cytometry. During bone marrow cell reconstitution, recipient mice were given antibiotic water (neomycin, 1 mg/mL) twice a week for a fortnight.

### RNA-seq

NK cells were sorted from spleens of *Stk11* ^*fl/fl*^ (WT) and *Stk11*^*fl/fl*^*CD122*^*Cre*^ (KO) mice using BD FACS Aria II (BD Bioscience). Total RNA was extracted using the RNeasy Micro Kit (Qiagen 74004) according to the manufacturer’s instructions. RNA purity and quantification were evaluated using the NanoDrop 2000 spectrophotometer (Thermo Scientific, USA). RNA integrity was assessed using the Agilent 2100 Bioanalyzer (Agilent Technologies, Santa Clara, CA, USA). Then the libraries were constructed using Single Cell Full Length mRNA-Amplification Kit (Vazyme, N712-03, Nanjing, China) and TruePrep DNA Library Prep Kit V2 for Illumina (Vazyme, TD502-02, Nanjing, China) according to the manufacturer’s protocols. The transcriptome sequencing and analysis were conducted by OE Biotech Co., Ltd. (Shanghai, China).

### Autophagy detection

The autophagosome was measured by Autophagy Detection Kit (Cat#D677, DOJIODO) according to the manufacturer’s instructions. Briefly, splenic cells from *Stk11*^*fl/fl*^ and *Stk11*^*fl/fl*^*CD122*^*Cre*^ mice were seeded in a total volume of 500 μL in a 48-well plate at a concentration of 2 × 10^6^ cells/mL and maintained for 4 h in a 37 °C incubator containing 5% CO_2_ plus DMSO or lysosomal inhibitor (Chloroquine, 30 μM). Cells were washed in Assay Buffer and stained with Autophagy Detection Kit and primary conjugated antibodies against different surface markers for 30 min at 37 °C. After washing with the wash buffer, cells were analyzed by flow cytometry.

### In vivo treatment

Three-week-old *Stk11* ^*fl/fl*^ and *Stk11*^*fl/fl*^*CD122*^*Cre*^ mice received intraperitoneal injections of either rapamycin (LC Laboratories; 2 mg/kg body weight) or A-769662 (MCE; 25 mg/kg body weight) every other day for 3–4 weeks. Control mice were injected with an equivalent volume of vehicle solvent. The vehicle for both drugs consisted of 5% DMSO, 40% PEG300, 5% Tween 80, and 50% deionized water. Following the final injection, mice were euthanized, and organs were harvested for subsequent analysis.

### Detection of intracellular staining for IFN-γ

Adult *Stk11* ^*fl/fl*^ and *Stk11*^*fl/fl*^*CD122*^*Cre*^ mice (20 g body weight) were intraperitoneally injected with 200 μg poly (I:C), and splenocytes were collected after 18 h. For RMA-S stimulation, poly (I:C)-activated splenocytes (2 × 10^6^) were co-cultured with an equal number of RMA-S cells in a total volume of 500 μL in 24-well plates. For antibody stimulation, 24-well plates were coated with anti-NK1.1 at a concentration of 1 μg/mL overnight, and then 4 × 10^6^ poly (I:C)-activated splenocytes were seeded into 24-well plates. BD GolgiStop™ reagent (BD Biosciences) was used to inhibit intracellular protein transport. Four hours after stimulation, cells were harvested for the detection of intracellular IFN-γ expression. First, the cells were stained with anti-CD3 and anti-NKp46, and then fixed and permeabilized with BD Cytofix/Cytoperm™ Buffer (BD Biosciences). These cells were then stained with anti-IFN-γ. Data were acquired using a CYTEK NORTHERN LIGHTS flow cytometer and analyzed using Flowjo software.

### In vivo rejection assay

Splenocytes derived from WT and *β2m*^−/−^ mice were subjected to red blood cell depletion using Ficoll-Hypaque density gradient centrifugation. Subsequently, the splenocytes obtained from *β2m*^−/−^ mice were labeled with 10 μM CFSE, while cells from WT mice were labeled with 1 μM CFSE (with a 10-fold lower concentration compared to *β2m*^−/−^ cells) using molecular probes. These two types of CFSE-labeled splenocytes were mixed at a 1:1 ratio. The resulting mixture, comprising 2 × 10^6^ splenocytes, was intravenously injected into the indicated mice that had been pre-treated with poly (I:C) at a dosage of 10 μg/g. After an interval of 18 h, flow cytometry was employed to assess the presence of CFSE-high cells in the spleens and lymph nodes. To ensure unbiased assessment, all flow cytometry samples were coded and analyzed by an investigator blinded to the experimental groups. The rejection ratio is calculated based on the following formula: Rejection (%) = 100*(β2m^+/+^ − β2m^−/−^) / (β2m^+/+^).

### In vivo RMA-S clearance assay

Mice pre-treated with poly (I:C) (10 μg/g) were intraperitoneally injected with a mixture of target cells consisting of NK-sensitive RMA-S cells expressing GFP (10^6^ cells) and NK-non-sensitive RMA cells expressing Ds-Red (10^6^ cells). After an additional 18-h period post-injection, the mice were euthanized, and cells present in the peritoneal cavity were collected through repeated washing with PBS supplemented with 2 mM EDTA. All samples were coded and analyzed by flow cytometry by an investigator blinded to the experimental groups to determine the relative percentages of RMA-S and RMA cells. Data from mice in which the injection failed (e.g., misdirected injection into the intestine) were excluded from the analysis. The rejection ratio was calculated based on the following formula: Rejection (%) = 100 × (RMA − RMA-S) / RMA.

### B16 melanoma lung metastasis mouse model

Wild-type and knockout littermate mice (8 weeks old) were PCR-genotyped and co-housed. B16F10 melanoma cells were intravenously injected (2 × 10^5^ per mouse). Mice were euthanized on day 14 post-injection, and lungs were collected and weighed. On the basis of stratification and randomized grouping, we also implemented a blinded analysis for metastatic nodule counting to avoid bias. All lung tissues were first coded by one researcher to hide genotype information, followed by nodule counting by another researcher who was unaware of the grouping (blinded to genotype). No mice met the pre-established exclusion criteria (e.g., technical injection failure or non-tumor-related death).

### Statistical analysis

Statistical analyses were performed using Prism 10 software (GraphPad, version 10.3.1). Differences between samples were analyzed using a two-tailed unpaired Student’s *t* test following confirmation of homogeneity of variance. Comparisons between three or more independent groups were performed using one-way ANOVA, followed by Tukey’s test for multiple comparisons. Two-way ANOVA was employed for analyses involving two independent categorical variables, followed by post hoc tests for multiple comparisons. Data are presented as mean ± SD. Specific statistical details (including exact P values, 95% confidence intervals) are given in Table S1 in the supplementary material.

## Supplementary information

supplemental materials

Related Manuscript File

## Data Availability

Data will be made available on request.
