# Supplementary material for: Liver kinase B1 maintains natural killer cell survival by regulating redox homeostasis
Source: Cell Death Dis. 2026 Mar 27;17(1):413. doi: 10.1038/s41419-026-08629-w (PMC13144693; doi:10.1038/s41419-026-08629-w)
Supplement: Supplementary file 1 — supplemental materials [file 41419_2026_8629_MOESM1_ESM.docx]

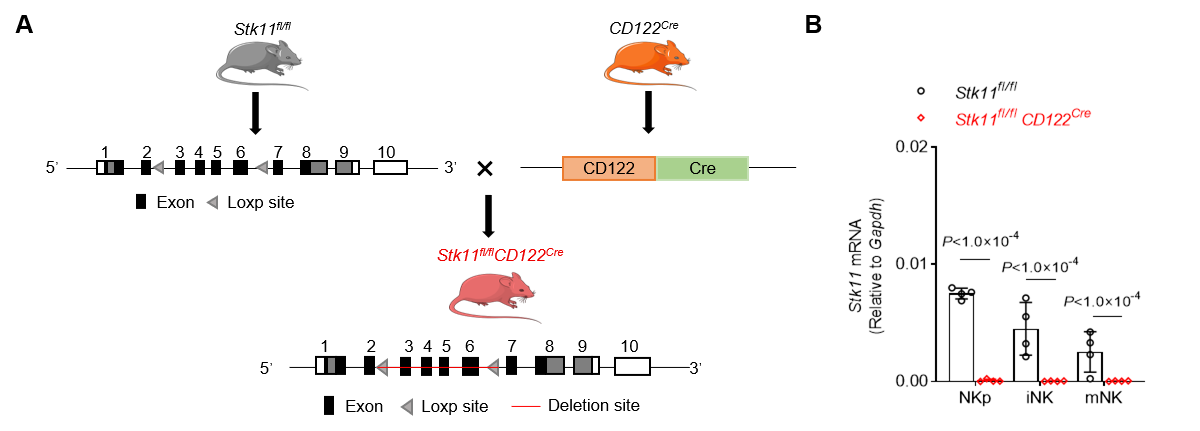


**Figure S1. Breeding strategies and the assessment of Lkb1 deletion efficiency in conditional targeting mouse models. (A)** Strategies for breeding *Stk11^fl/fl^CD122^Cre^* mice to achieve stage-specific Lkb1 ablation at the NK progenitor (NKp) stage. **(B)** Quantitative PCR analysis of *Stk11* expression in NKp, iNK, and mNK cells isolated from the bone marrow of *Stk11^fl/fl^* and *Stk11^fl/fl^CD122^Cre^* mice (n = 4). Expression levels were normalized to *Gapdh*. Data are presented as mean ± SD and representative of two (B) independent experiments with consistent results.


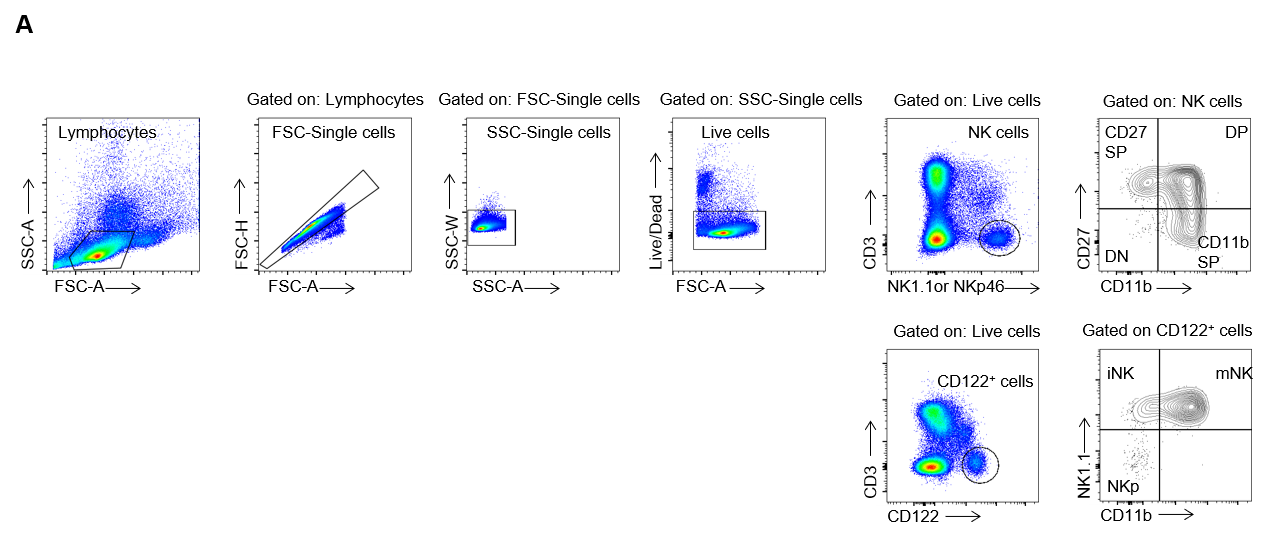


**Figure S2. Flow cytometry analysis strategy for NK cell proportion and development process. (A)** Gating strategies for NK cell population and developmental stages.


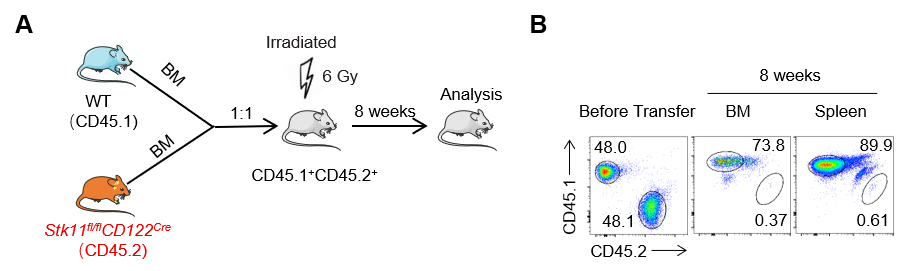


**Figure S3. Construction of bone marrow chimera mice and assessment of NK cells in the recipient mice. (A)** Schematic diagram of the construction of bone marrow chimera mice. **(B)** Representative flow cytometry plots of NK cells in the bone marrow (BM) and spleen from the recipient mice. The percentages of injected cells are shown.


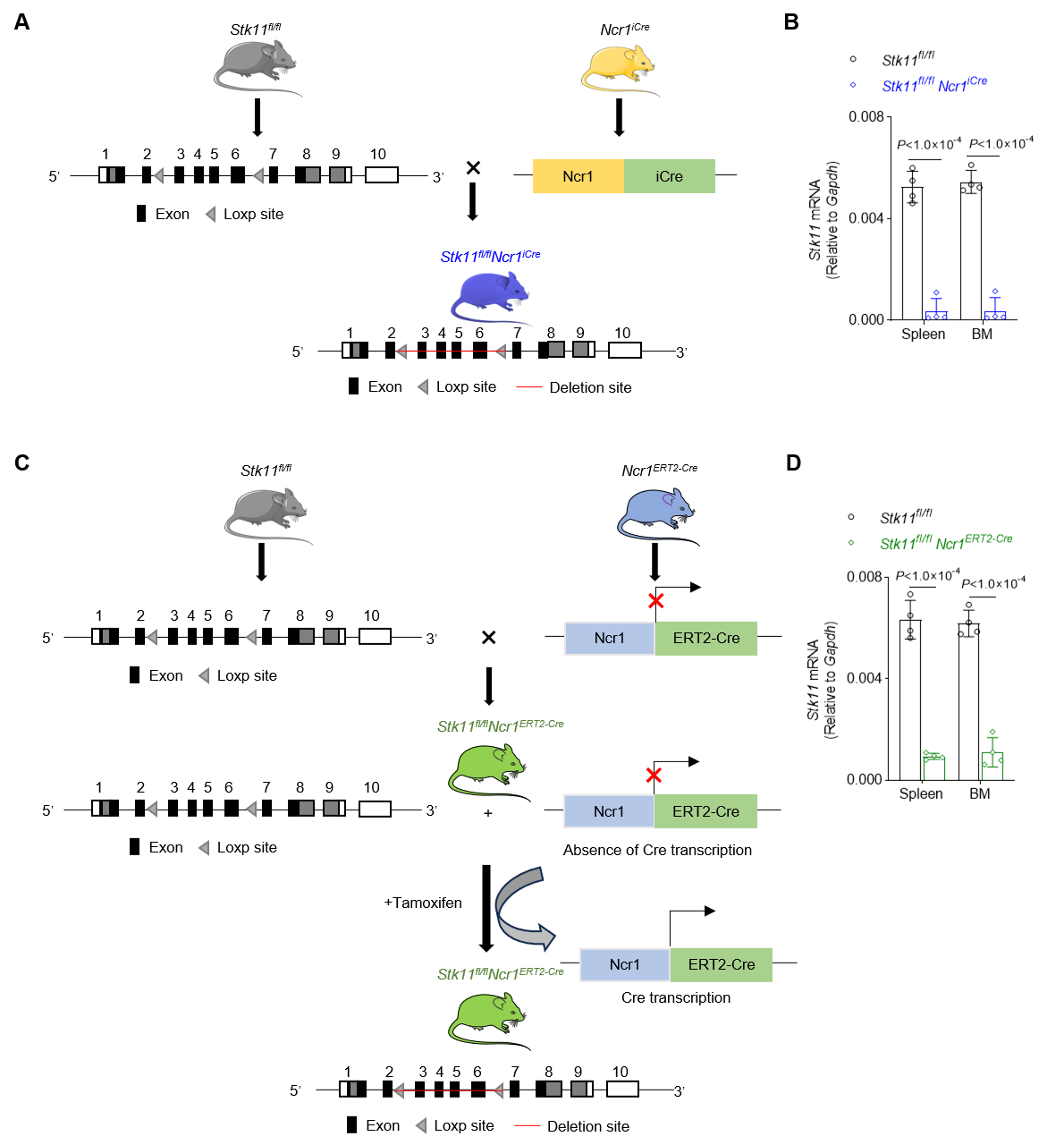


**Figure S4. Strategies for constructing and assessing Lkb1 deletion efficiency in mouse models. (A)** Breeding strategies for the *Ncr1-iCre*-mediated NK cell-specific conditional Lkb1 knockout mouse model. **(B)** Quantitative PCR analysis of *Stk11* expression in NK cells from the spleen and bone marrow (BM) of *Stk11^fl/fl^* and *Stk11^fl/fl^Ncr1^iCre^* mice (n = 4). **(C)** Breeding strategies for the *Ncr1-ERT2-Cre*-mediated NK cell-specific inducible Lkb1 knockout mouse model. **(D)** Quantitative PCR analysis of *Stk11* expression in NK cells from the spleen and BM of tamoxifen-induced *Stk11^fl/fl^* and *Stk11^fl/fl^Ncr1^ERT2-Cre^* mice (n = 4). B and D, Data are presented as mean ± SD and are representative of two (B, D) independent experiments with consistent results.


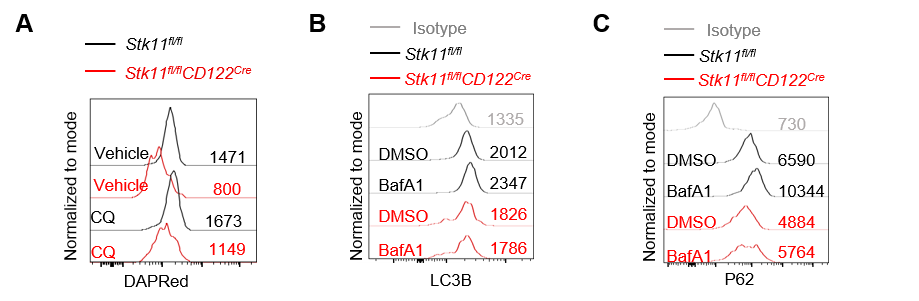


**Figure S5. Loss of Lkb1 in NK cells impairs autophagic processes.** (**A-C**) Representative histograms of DAPRed (A), LC3B (B) and P62 (C) in NK cells from the spleens of *Stk11^fl/fl^* and *Stk11^fl/fl^CD122^Cre^* mice, treated with or without BafA1 for 6 hours (n = 4).


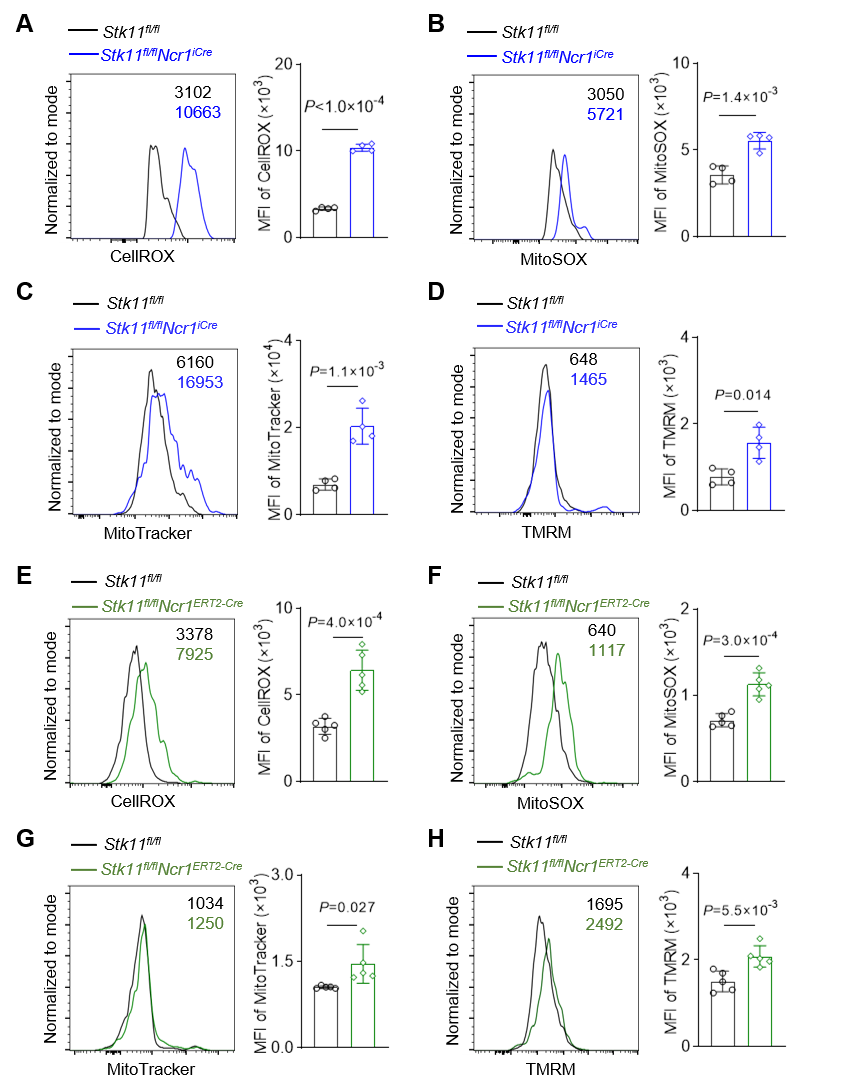


**Figure S6. Loss of Lkb1 in NK cells impairs mitochondrial function and promotes ROS expression. (A-D)** Representative histograms (left) and mean fluorescence intensity (MFI) quantification (right) of CellROX (A), MitoSOX (B), MitoTracker (C), and TMRM (D) levels in NK cells from the spleens of *Stk11^fl/fl^ and Stk11^fl/fl^Ncr1^iCre^* mice (n = 4). **(E-H)** Representative histograms (left) and MFI quantification (right) of MitoTracker (E), TMRM (F), CellROX (G), and MitoSOX (H) levels in NK cells from the spleens of tamoxifen-induced *Stk11^fl/fl^ and Stk11^fl/fl^Ncr1^ERT2-Cre^* mice (n = 5). Data are presented as means ± SD and representative of three independent experiments. Two-tailed Student t test (A-H).


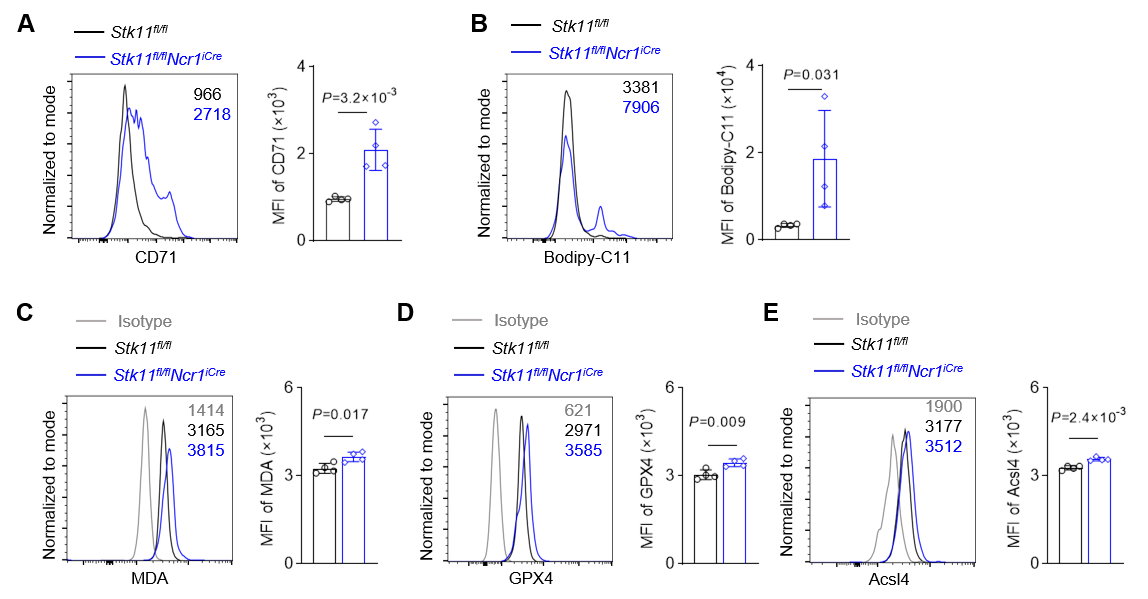


**Figure S7. Iron death pathway is impaired in LKB1-deficient NK cells. (A-B)** Representative histograms (left) and MFI quantification (right) of CD71 (A) and Bodipy-C11 (B) levels in NK cells from spleens of *Stk11^fl/fl^ and Stk11^fl/fl^Ncr1^iCre^* mice (n = 4). **(C-E)** Representative histograms (left) and MFI quantification (right) of MDA (C), GPX4 (D), and Acsl4 (E) levels in NK cells from the spleens of tamoxifen-induced *Stk11^fl/fl^ and Stk11^fl/fl^Ncr1^iCre^* mice (n = 4). Data are presented as means ± SD. Two-tailed Student t test(A-E). Data are representative of three independent experiments with consistent results.


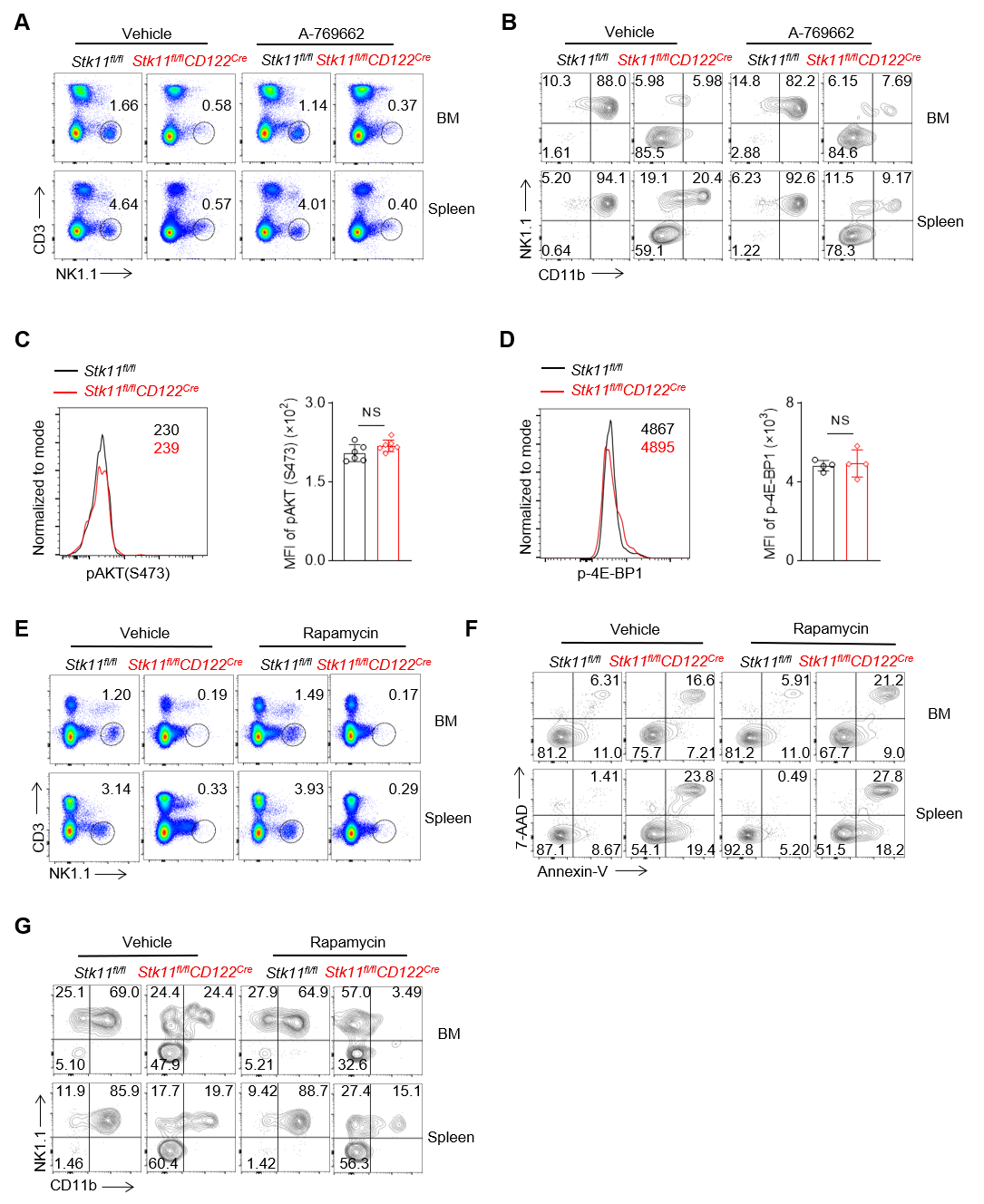


**Figure S8. Pharmacologic AMPK activation or mTORC1 inhibition could not restore the homeostatic and developmental defects of NK cells caused by Lkb1 deficiency.**  **(A)** Representative flow cytometry plots of NK cells in the bone marrow (BM) and spleen from *Stk11^fl/fl^ and Stk11^fl/fl^CD122^Cre^* mice, treated with or without A-769662. **(B)** Representative flow cytometry plots of NKp, imNK, and mNK cells in the BM and spleen from *Stk11^fl/fl^ and Stk11^fl/fl^CD122^Cre^* mice, treated with or without A-769662. **(C, D)** Representative histograms (left) and MFI quantification (right) of p-AKT(S473) (C), and p-4E-BP1 (D) levels in NK cells from the spleens of *Stk11^fl/fl^ and Stk11^fl/fl^CD122^Cre^* mice (n = 4). **(E)** Representative flow cytometry plots of NK cells in the BM and spleen from *Stk11^fl/fl^ and Stk11^fl/fl^CD122^Cre^* mice, treated with or without Rapamycin. **(F)** Representative flow cytometry plots of Annexin V-positive NK cells in the BM and spleen from *Stk11^fl/fl^ and Stk11^fl/fl^CD122^Cre^* mice, treated with or without Rapamycin. **(G)** Representative flow cytometry plots of NKp, imNK, and mNK cells in the BM and spleen from *Stk11^fl/fl^ and Stk11^fl/fl^CD122^Cre^* mice, treated with or without Rapamycin. Two-tailed Student t test (C, D) Data (C, D) are presented as means ± SD and compiled from three independent experiments.


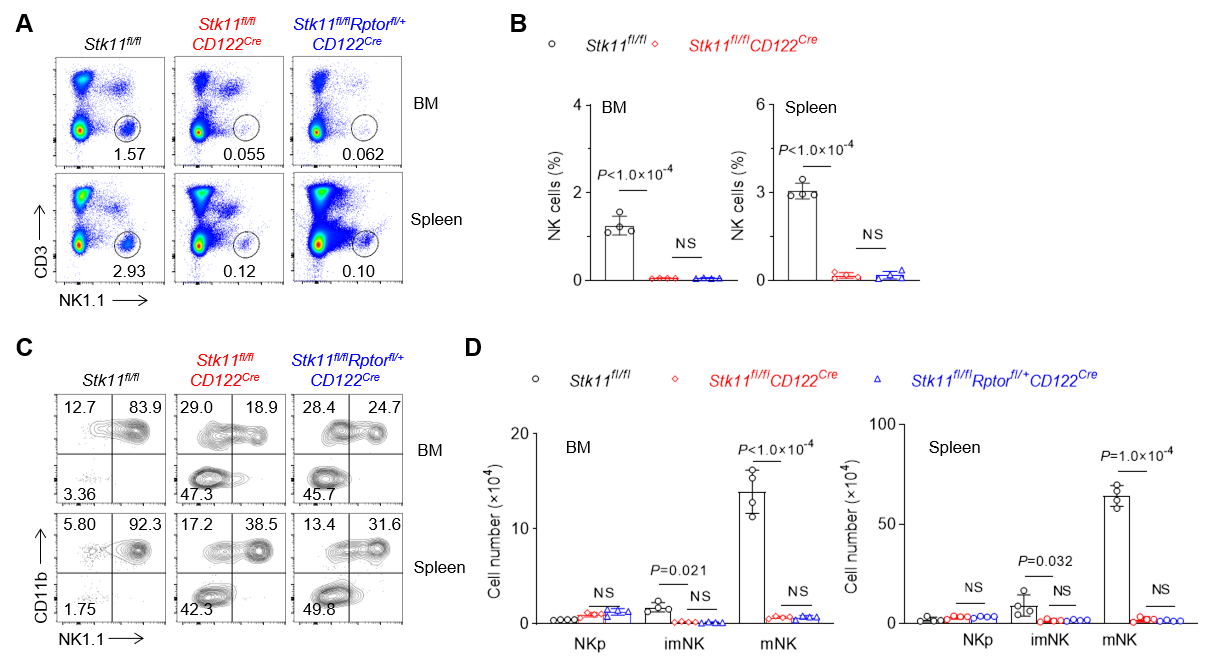


**Figure S9. Raptor inactivation does not restore NK cell numbers and development in Lkb1 deficiency. (A, B)** Representative flow cytometry plots (A) and percentages (B) of NK cells (CD3^−^NK1.1^+^) in the BM and spleen from *Stk11^fl/fl^*, *Stk11^fl/fl^CD122^Cre^* and *Stk11^fl/fl^Rptor^fl/fl^CD122^Cre^* mice (n = 4). **(C-D)** Representative flow cytometry plots (C), and absolute number quantification (D) of NKp, imNK, and mNK cells in the BM and spleen from *Stk11^fl/fl^*, *Stk11^fl/fl^CD122^Cre^* and *Stk11^fl/fl^Rptor^fl/fl^CD122^Cre^* mice (n = 4). Data are presented as means ± SD. Two-tailed Student t test (B, D). Data are compiled from two (A-D) independent experiments.


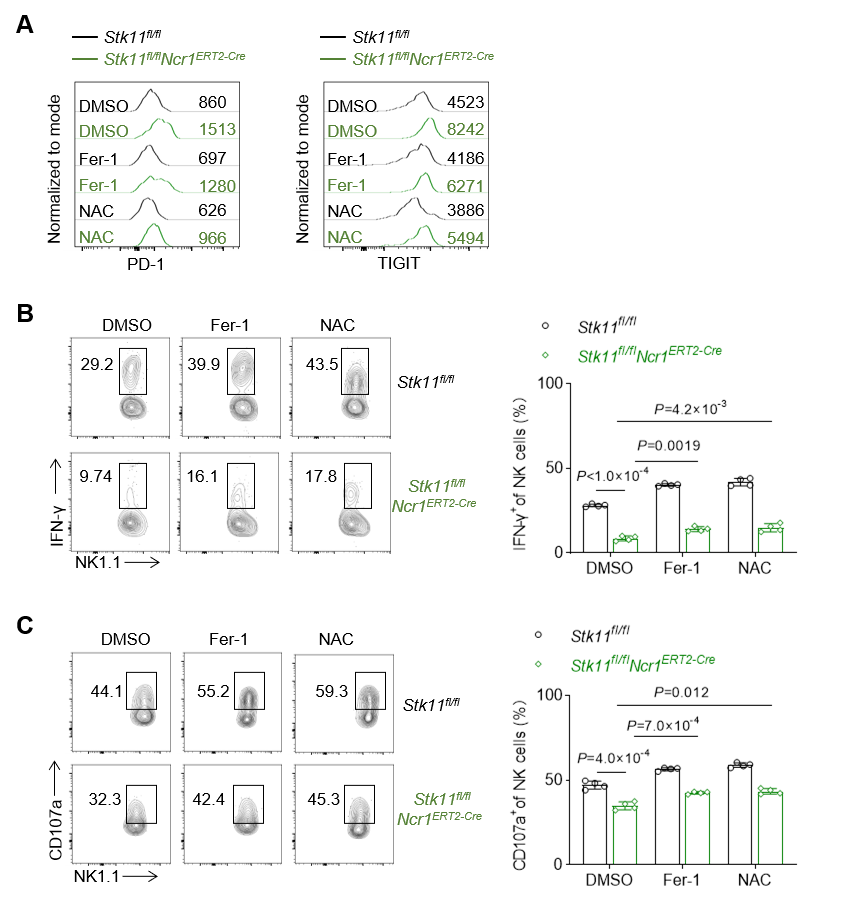


**Figure S10. NAC and Fer-1 alleviate the exhausted phenotype and functional impairment of LKB1-deficient NK cells.** **(A)** Representative histograms of PD-1 (left) and TIGIT (right) levels in spleens of Stk11^fl/fl^ and *Stk11^fl/fl^Ncr1^ERT2-Cre^* mice treated with NAC or Fer-1 for 24h (n = 4). **(B, C)** Representative flow cytometry plots (left) and percentage (right) of IFN-γ⁺ (B) and CD107a⁺ (C) NK cells from *Stk11^fl/fl^* and *Stk11^fl/fl^Ncr1^ERT2-Cre^* mice after anti-RMA-S stimulation, with or without pre-treatment of NAC and Fer-1 (n = 4). Data are presented as means ± SD. Two-way ANOVA (B, C). Data are compiled from two independent experiments.
